# Electrochemical and DFT studies of *Terminalia bellerica* fruit extract as an eco-friendly inhibitor for the corrosion of steel

**DOI:** 10.1038/s41598-023-45283-0

**Published:** 2023-11-08

**Authors:** Ahmed M. Abuelela, Jasdeep Kaur, Akhil Saxena, Mahmoud A. Bedair, Dakeshwar Kumar Verma, Elyor Berdimurodov

**Affiliations:** 1 Department of Chemistry, College of Science, King Faisal University, Al Ahsa, Saudi Arabia; 2 Department of Chemistry, Faculty of Science, Al-Azhar University, Nasr City, Cairo 11884 Egypt; 3 Department of Chemistry, Chandigarh University, Mohali, India; 4 Department of Chemistry, College of Science, University of Bisha, P.O. Box 511, 61922 Bisha, Saudi Arabia; 5 Department of Chemistry, Government Digvijay Autonomous Postgraduate College, Rajnandgaon, Chhattisgarh 491441 India; 6 Chemical & Materials Engineering, New Uzbekistan University, 100007 Tashkent, Uzbekistan; 7 Medical School, Central Asian University, 111221 Tashkent, Uzbekistan

**Keywords:** Electrochemistry, Materials chemistry, Physical chemistry, Theoretical chemistry

## Abstract

It is well known that metal corrosion causes serious economy losses worldwide. One of the most effective ways to prevent corrosion is the continuous development of high-efficient and environment-friendly corrosion inhibitors. Among the widely used organic and inorganic corrosion inhibitors, plant extracts are top candidates due to their nontoxic nature. The present study reports a novel application of the methanolic extract of *Terminalia bellerica* fruits as an environment friendly corrosion inhibitor for steel in sulphuric acid medium. The phytochemicals of the extract, namely Ellagic, Gallic, and Malic acids, play a key role of the anti-corrosive behavior of the extract. The corrosion prevention activity was studied on the steel in 1 M H_2_SO_4_ using a variety of approaches including weight loss analysis (WL), scanning electron microscope (SEM), electrochemical impedance spectroscopy (EIS), density functional theory (DFT), natural bond orbital analysis (NBO), Fukui function and Monte Carlo simulations (MC). In 1 M H_2_SO_4_ solution, the maximum electrochemical inhibition efficiency of 91.79% was observed at 4000 mg/L concentration of the extract. The NBO analysis showed that the charge density of the double bonds and the oxygen atoms of carbonyl and hydroxyl groups of the phytochemicals lies on the top of the natural bond orbitals which promotes the anticorrosive properties of the investigated inhibitors. The surface coverage of steel was validated by SEM measurements. According to DFT studies, numerous nucleophilic regions were present in the active phytochemical constituents of the inhibitor, demonstrating their favorable nucleophilicity. The computed electronic structure of the phytochemicals revealed band gaps of 4.813, 5.444, and 7.562 eV for Ellagic, Gallic, and Malic acids respectively suggesting effective metal-inhibitor interactions. A good correlation between experimental and theoretical findings was addressed.

## Introduction

Steel is a preferred choice in variety of industries due to its great mechanical strength. However, in industries, the corrosion of steel occurs because of interaction with corrosive media like sulfuric acid and hydrochloric acid, which are commonly employed for cleaning and descaling purposes^1^. Corrosion can cause dangerous and expensive damage to everything from pipelines, bridges, and public buildings to vehicles, water and wastewater systems, and even home appliances^2^. It was indicted the iron rust can easily absorb the Arsenic content and contaminate the external environment^3^. In addition to the existence of the iron rust can enhance the growth rate of the Legionella bacteria in water. The growth rate can be enhanced by to 10^3^–10^5^ fold with the ferric oxide^4^. The employment of inhibitors is one of the most efficient strategies to inhibit unexpected metal dissolution, particularly in acid solutions^5^. A lot of synthetic chemicals are being used as corrosion inhibitors to reduce corrosion reaction, however many of them have significant environmental consequences and also hazardous to human health^6^. To overcome this problem, natural and biodegradable corrosion inhibitors must be employed due to their non-toxicity and affordability. Various phytochemicals are present in plant extracts, like terpenoids, alkaloids, flavonoids, coumarins, tannins and poly phenols that contain heteroatoms that effectively play role in protective layer formation on the steel surface^7–9^. Several plants are available from the literature having different phytochemicals as demonstrated in Table 1^10–17^.

**Table 1:** Different phytochemicals in plants.

S. No	Plant name	Phytochemicals	References
1	Echinacea root extract	Polysaccharides, caffeic acid derivatives, alkamides, and lipoproteins	^10^
2	*Papaver somniferum*	Narcotine, Papaverine and Thebaine	^11^
3	*Allium sativum*	Disulphide	^12^
4	*Sida cordifolia*	Vasicinone	^13^
5	*Saraca ashoka*	Epicatechin	^14^
6	*Cuscuta reflexa*	3-methoxy-3,4,5,7-tetrahydroxy flavones	^15^
7	*Armoracia rusticana*	Wine lactone	^16^
8	*Myristica fragrans*	Phenolic compound	^17^

The existence of phytochemicals boosts the effectiveness of plant as corrosion inhibitors. Corrosion of steel in aggressive media can be controlled by using such green inhibitors that comprise various heteroatoms like oxygen, nitrogen, and sulfur^18–23^.

Bhawsar et al.^24^ observed that the extract of *Nicotiana tabacum,* which include nicotine, inhibit steel in 2 M sulphuric acid, with efficiency of 94% in 1 g/L. *Rauwolfia macrophylla* was discovered as a corrosion protector in HCl as well as in H_2_SO_4_ because of the presence of alkaloids perakine and tetrahydroalastonine^25^. These phytochemicals suppress, or even prevent the corrosion of the steel when employed in acidic media. Numerous plant parts, having different phytochemicals were used as corrosion inhibitors and showed good inhibition efficiency as presented in Table 2^26–40^.

**Table 2:** Corrosion inhibition efficiency of some plants.

S. no.	Plant name	Plant Part	Efficiency/ Conc. of extract	Medium used	Phytochemicals	Reference
1	*Cryptostegia grandiflora*	Leaves	87% at 500 ppm	1 M H_2_SO_4_	Hydroxyl cinnamic acid	^26^
2	*Citrus aurantium*	Leaves	89% at 10,000 ppm	1 M H_2_SO_4_	Phenolic compound	^27^
3	*Chinese gosebery fruit*	Fruit shell	92% at 800 ppm	1 M HCl	Sucrose and maltose	^28^
4	*Borage flower*	Flower	91% at 800 ppm	1 M HCl	Lactic acid and nicotinic acid	^29^
5	*Parseley petroselium*	Leaves	92% at 5000 ppm	1 M HCl	Chromen-2-one	^30^
6	*Lannea coromandelica*	Leaf	93.8% at 2000 ppm	1 M H_2_SO_4_	Flavonoids	^31^
7	*Aloe Vera*	Leaves	96% at 300 ppm	1 M H_2_SO_4_	Anthrones	^32^
8	*Nauclea latifolia*	Root	94% at 4000 ppm	1 M H_2_SO_4_	Saponins	^33^
9	*Ficus racemose*	Stem	90.5%at 2500 ppm	1N H_2_SO_4_	Triterpenes, sterols, long chain fatty acids	^34^
10	*Cannabis sativa*	Leaves	97.31% at 200 ppm	0.5 M H_2_SO_4_	Cannabinoids	^35^
11	*Hemidesmus indicu*	Leaves	98.05 at 4000 ppm	1 M H_2_SO_4_	Alkaloids, tannins, saponins and steroids	^36^
12	*Coconut*	Coir dust	94.3% at 500 ppm	0.5 M H_2_SO_4_	Saponins, flavonoids, tannins, phlobatannins, polyphenols and anthraquinones	^37^
13	*Adhatoda vasica*	Leaves and flower	98.9% at 3000 ppm	0.5 M H_2_SO_4_	Vasicine and vasicinone	^38^
14	*Citrullus lanatus*	Fruit	91% at 800 ppm	1 M HCl	Citrulline and nesperetin	^39^
15	*Eucalyptus*	Leaves	88% at 800 ppm	1 M HCl	Eucalyptone and ellagic acid	^40^

**Figure 1:**
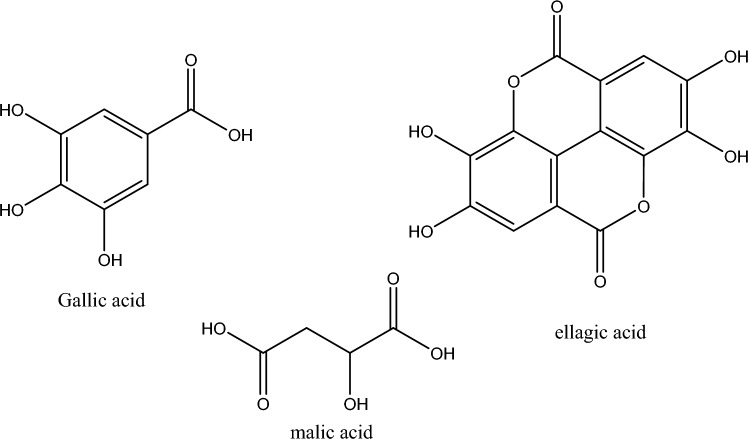
Phytochemicals constituents of *Terminalia bellerica*.

The main vision of the current research is to determine the inhibitory effect of fruit extract of *Terminalia bellerica*. This extract can be used as a pickling agent to reduce the corrosion of steel in industries where acid corrosion occurs. *Terminalia bellerica* also known as baheda that is a species of *Combretaceae* and a huge deciduous tree that can be found on the plains and on the lower slopes. Medicinal properties of the *Terminalia bellerica* fruit have been studied earlier; however, anticorrosive properties were not investigated yet. A literature survey reveals that *Terminalia bellerica* extract contains Ellagic, Gallic, and Malic Acid^41,42^ as displayed in Fig. 1. The following study include electrochemical measurements, surface morphology measurements, DFT calculations and Monte Carlo simulations.

## Experimental studies

### Specimen preparation

The steel alloy composition used in the study is depicted in Table 3. The steel sample has a 1 cm^2^ surface area. Each steel coupon was cleaned using several sandpapers prior to the corrosion examination.

**Table 3:** Composition of the studied steel coupons.

S. no.	Metals present	% composition
1	Iron	99.2
2	Silicon	0.120
3	Carbon	0.105
4	Manganese	0.378
5	Phosphorus	0.079
6	Sulphur	0.0798

### Sample collection and preparation of an inhibitor

Plants (either cultivated or wild), including the collection of plant material, complied with relevant institutional, national, and international guidelines and legislation. *Terminalia bellerica* fruit sample was purchased from the market of Sirhind, Punjab. The dried fruits were then crushed to a powder using a grinder. Then 250 g powdered fruits were refluxed at 50 °C for around 24 h using a Soxhlet apparatus and 250 ml of methanol. A hot water bath was then utilized to dry the extract. The concentration range of the *Terminalia bellerica* fruit extract employed was (1000–4000 mg/l) in 1 M H_2_SO_4_ solution.

### Preparation of the corrosive media

For the current experiment, we utilized a 1 M H_2_SO_4_ solution that was made using AR grade sulphuric acid from Loba Chemie in double-distilled water. To get various extract concentrations, it was then further diluted.

### Weight-loss measurement

Before every corrosion study, the 1 cm^2^ steel coupons were polished with emery paper of various grades. The steel coupons were firstly weighed and then submerged in 1 M sulphuric acid with several inhibitor concentrations (1000, 2000, 3000, and 4000 mgL^-1^) for 24 h. The coupons of steel were taken out after being exposed to acidic media. Following acetone rinsing and drying of the coupons, the weight loss data were computed using the following equation^43,44^:

1 $$\eta {\%} = \frac{W_0-W_i}{W_0}\times 100$$

2 $$\theta = \frac{W_0-W_i}{W_0}$$

$W_0-W_i=\Delta W$ is the weight loss (mg), where w_0_ and w_i_ is the loss of weight without inhibitor and with involvement of inhibitors, respectively and θ is the surface coverage.

### Electrochemical studies

Electrochemical analysis (PDP and EIS) of the steel coupons was performed with a Metrohm Autolab electrochemical analyzer^45,46^. There were three electrodes connected in the corrosion cell: a working electrode made of steel, a reference electrode made of saturated calomel, and a counter electrode made of platinum. The steel was covered with araldite resin, leaving a 1 cm^2^ active region exposed. Current–potential curves were obtained by varying the electrode potential between −250 and + 250 mV in relation to the open circuit potential^45,46^.

The following relationship is used to calculate efficiency^47^:

3 $$\eta \%= \frac{{I}_{0corr}-{I}_{icorr}}{{I}_{0corr}} \times 100$$

I_0corr_ and I_icorr_ are measurements of the corrosion current density without and with plant extract. The same electrochemical workstation was employed for PDP analysis as well as for EIS measurements. To set the OCP, the steel electrode was immersed in the acidic medium for approximately 45 min prior to each experiment. The effectiveness of the inhibition was evaluated using the following equation^48^:

4 $$\eta \%= \frac{{R}_{ct}-{{R}^{0}}_{ct}}{{R}_{ct}} \times 100$$

R^0^_ct_ is used to demonstrate the charge transfer resistance of a solution without any plant extract in it, and R_ct_ that refers to the charge transfer resistance of a solution containing a plant extract.

### Phytochemical testing

Methanolic extract of *Terminalia bellerica* was examined for the phytochemicals like alkaloids, flavonoids, saponins, sugar, coumarin, and quinones. A testing solution was prepared by dissolving 0.35 gm of the concentrate in 50 mL of methanol which was subsequently utilized for the phytochemicals investigated. Numerous studies were carried out to assess the phytochemical conformation^41^.

### UV–visible spectra analysis

Using a UV–Visible spectrophotometer, the spectrum of a *Terminalia bellerica* extract in 1 M H_2_SO_4_ was determined. To describe the adsorption/desorption behavior of the inhibitor, UV/Visible analysis was conducted for the solutions in which steel specimen was dipped for 24 h and for solutions in which steel specimen was not dipped. To understand the inhibitory mechanism, both spectra was assigned^49^.

### Surface inspection

SEM and AFM data of steel were utilized to estimate the surface morphology during the corrosion-protection process. The SEM and AFM pictures of cleaned steel specimen and steel that was dipped in an acid solution without and with an inhibitor were examined.

### Theoretical models

**Figure 2:**
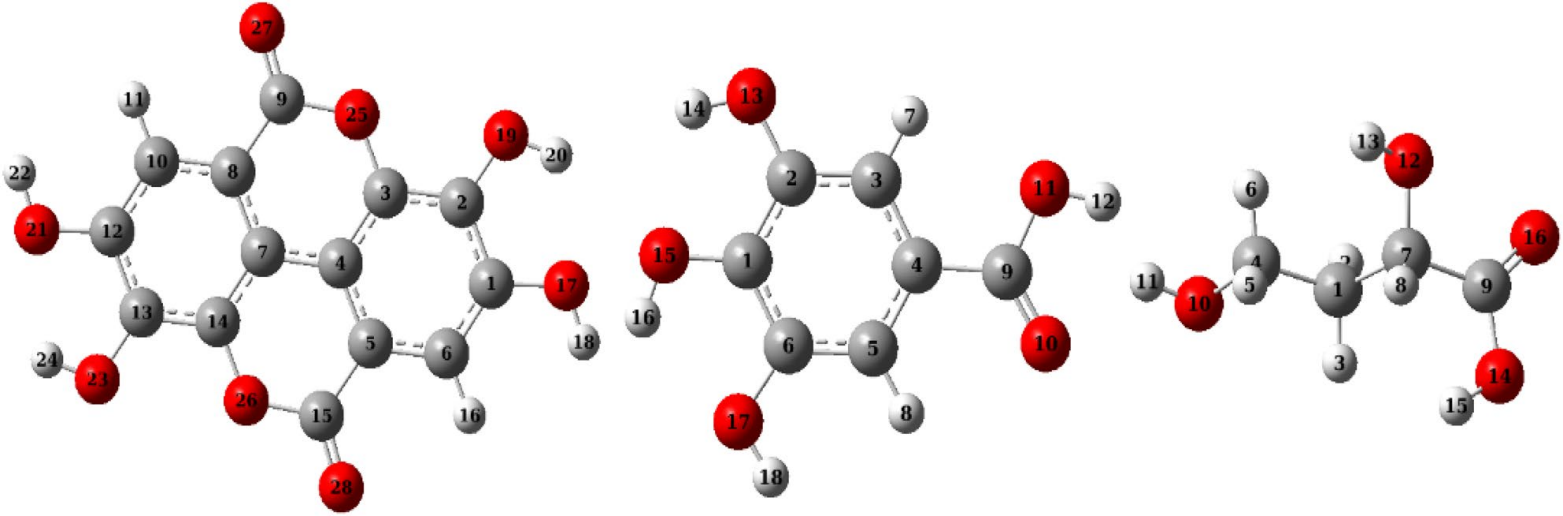
Optimized structural parameters at B3LYP/6-31g(d,p): from left (ellagic, gallic and malic acids).

Initial guesses of structures of Ellagic, Gallic, and Malic acids were prepared by ChemBioDraw Ultra 14.0 in 2D dimensions then a minimization (energy/geometry) was performed by Gaussian 09 revison-A.02-SMP package^50^ at B3LYP/6-31 g(d,p) level^51–53^. The optimized parameters were reached after achieving the convergence criteria indicated by Gaussian 09: the maximum remaining force on an atom and the average root mean square (RMS) force on all atoms are below the tolerance threshold $45\times {10}^{-5}$ and $30\times {10}^{-5}$ Hartree, respectively as well as the maximum structural drift of one coordinate and RMS change over all structural parameters in the last two iterations are below $18\times {10}^{-4}$ and $12\times {10}^{-4}$ Å , respectively. Obtained optimized molecules representing local minima were sketched by Gauss view 6 and presented in Fig. 2^54^.

Often Density Functional Theory (DFT) calculations underestimate Frontier molecular orbitals eigen values^55^, therefore the DFT calculations were repeated using the Time-dependent density-functional theory (TD-DFT) method to calculate the lowest excited state energy and therefore correct the value of band gap energy ${\Delta E}_{gap}$ between Highest Occupied Molecular Orbital (HOMO) and Lowest Unoccupied Molecular Orbital (LUMO). Six energy states were included for this calculation at TD-B3LYP/6-31 g(d,p) following the procedure described by Ref.^55^. ${E}_{HOMO} \mathrm{and} {E}_{LUMO}$ as well as their corresponding reactivity descriptors were obtained as described by Ref.^56^. HOMO and LUMO surfaces with an iso value of 0.02 and density of 0.0004 were presented as transparent surfaces using the molecular orbital editor of Gauss view 6^57^. Natural bond orbitals (NBOs) were measured at B3LYP/6-31 g(d,p) level for selected sites of Ellagic, Gallic, and Malic acids. NBOs surfaces were created using the NBO Version 3.1^58^ in Gaussian 09 software and plotted using the same isovalue of 0.02 and density of 0.0004 in order to compare them to Frontier Molecular Orbitals (FMOs). In addition, Fukui functions were calculated by addition of a positive and a negative charge to the neutral molecules and then re-optimizing it at B3LYP/6-31g (d,p).

Including water as a solvent, (2H^+^ and SO_4_^--^) as corrosive medium (200 H_2_O, 20 H_3_O^+^, 10 SO_4_^--^), a Monte Carlo simulation had been used to get deeper details of the adsorption pattern for the *Terminalia bellerica* fruit extract phytochemical structures and their binding with the metal surface. The simulation was done using the Adsorption Locator module included in the Materials Studio 7.0 program^59^. All constituents within the structure were optimized using a COMPASS force field. Fe (1 1 0) unit cell with 11 layers of (11 × 11) atoms (under periodic boundary conditions with size 18.386 × 22.341 × 22.341 Å) and a vacuum slape of 30 Å which was employed to depict the steel surface. The electrostatic energy was determined using the Ewald summation technique, whereas the van der Waals energy was calculated utilizing the atom-based procedure^60^.

## Result and discussions

### Measurements of weight reduction

**Figure 3:**
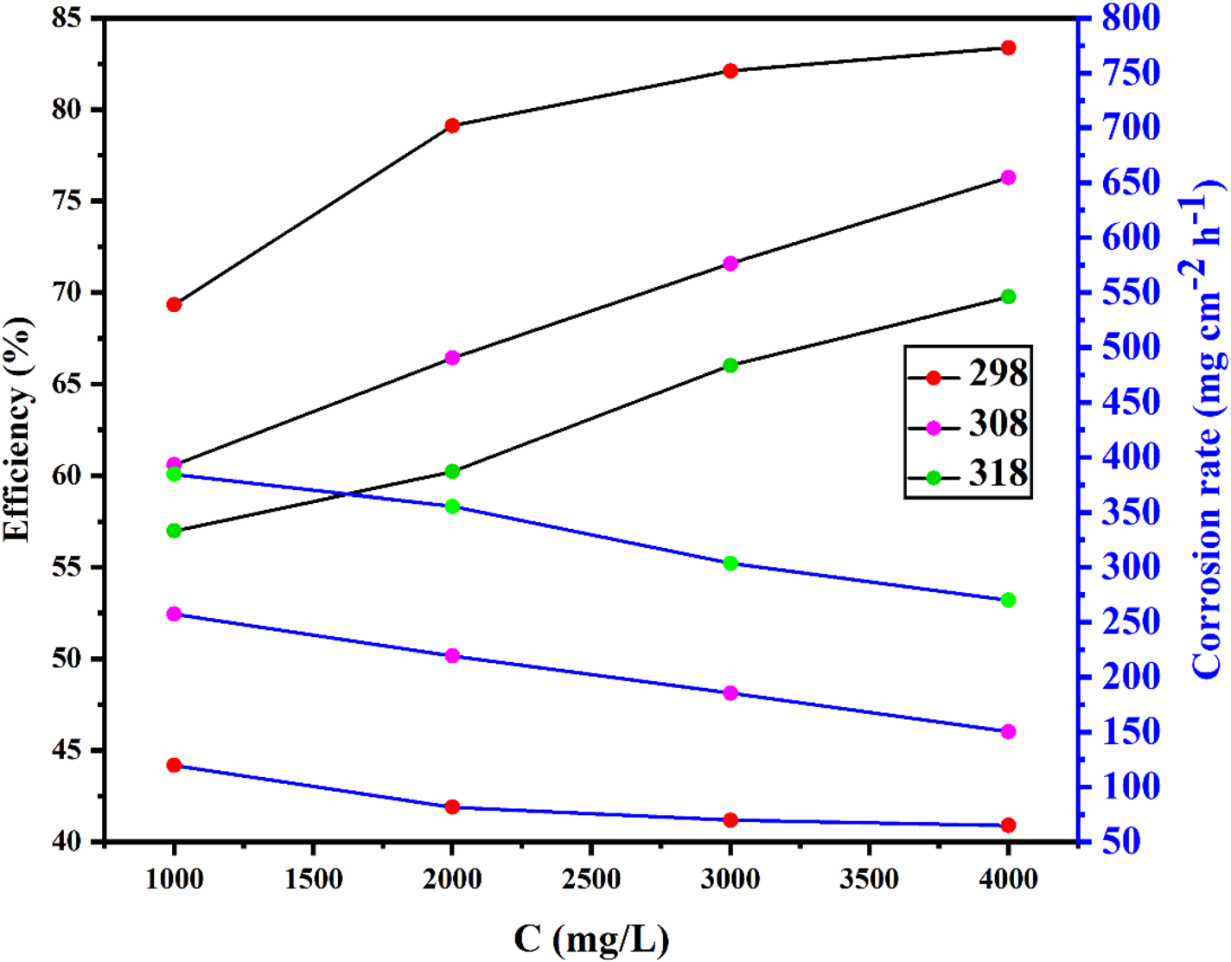
Corrosion inhibition efficiency of *Terminalia bellerica* extract at different concentrations.

Weight loss values, surface coverage and inhibitory effectiveness were determined and presented in Table 4 at varying concentrations of *Terminalia bellerica* extract at different environment temperatures. According to the information in the Table 4, by raising the extract's concentration, inhibition effectiveness increases (approached a maximum value of 83.38% at 4000 ppm and 298 K) while the corrosion rate decreased, which can also be inferred from the plot in Fig. 3. The corrosion rate was calculated using the following equation^61^:

5 $$Corrosion\,rate (mpy)= \frac{k x W}{D x A x T}$$

where *K* is the corrosion constant, *W* is the loss of weight after corrosion (mg), *A* is the total area of the coupon (cm^2^), *T* is the corrosion time (h) and *D* coupon density (g/cm^3^). This increase in inhibition effectiveness is only possible if electron-rich hereto atoms were adsorbed on the steel surface so that it retards the rate of metal corrosion in the abrasive medium^62,63^.

**Table 4:** Weight loss data of inhibition efficiency and surface coverage of *Terminalia bellerica* in 1 M H_2_SO_4._

Corrosive medium	Temp (K)	Conc. (mg/L)	Weight loss (mg)	Corrosion rate	Efficiency (%)	Surface coverage (θ)
1 M H_2_SO_4_	298	0	3.419	390.544	–	–
	298	1000	1.048	119.711	69.34	0.6934
	298	2000	0.714	81.559	79.11	0.7911
	298	3000	0.611	69.793	82.12	0.8212
	298	4000	0.568	64.881	83.38	0.8338
	308	0	5.718	653.153	–	–
	308	1000	2.254	257.469	60.58	0.6058
	308	2000	1.920	219.317	66.42	0.6642
	308	3000	1.624	185.506	71.59	0.7159
	308	4000	1.315	150.209	76.28	0.7628
	318	0	7.824	893.717	–	–
	318	1000	3.365	384.376	56.99	0.5699
	318	2000	3.112	355.476	60.22	0.6022
	318	3000	2.659	303.731	66.01	0.6601
	318	4000	2.365	270.148	69.77	0.6977

As the temperature ascending from 298 to 318 K, the rate of corrosion was raised which led to a decline in protection ability (Table 4, Fig. 3). Physical adsorption may be responsible for the decline in protective efficacy with raising temperature at all the evaluated doses. Due to desorption, the inhibitor molecules get detached from the steel substrate at elevated temperatures^43^.

### Adsorption isotherm

**Figure 4:**
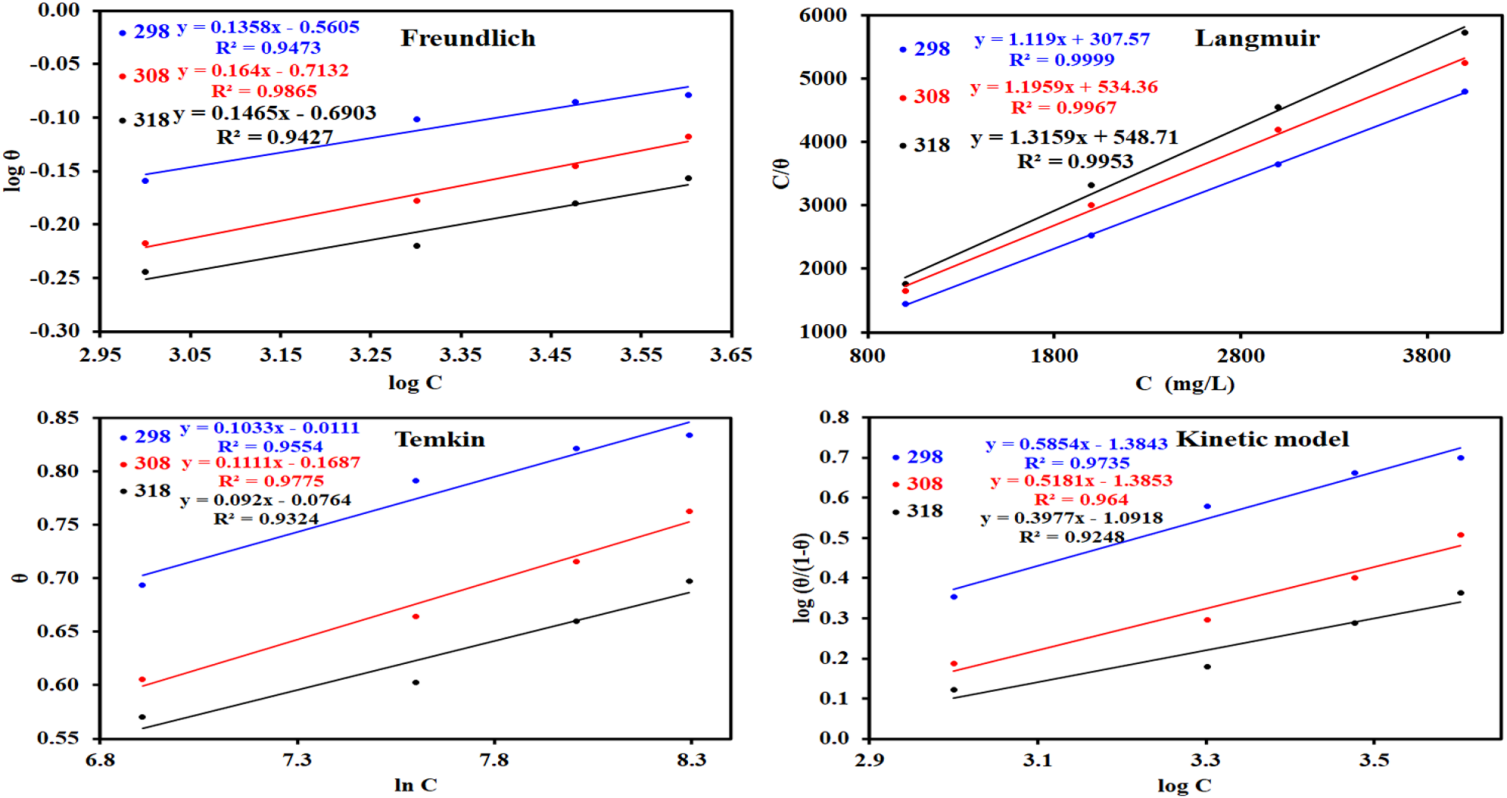
Adsorption isotherms for *Terminalia bellerica* on steel in 1 M H_2_SO_4_.

The data calculated by weight loss analysis can be fitted to different isotherms to examine the adsorption characteristics^64^. The fitting results were collected for four different isotherms (Freundlich, Langmuir, Temkin and kinetic-thermodynamic models) in Table 5 and Fig. 4. Langmuir model showed high fitting affinity to the WL data (R^2^ = 0.99986 at 298 K, 0.99669 at 308 K and 0.99535 at 318 K)^65^. By plotting C/θ vs. log C, Eq. (6) can be employed to calculate the adsorption^66^:

**Table 5:** Adsorption isotherms models of the *Terminalia bellerica* with values of R^2^, slopes, intercepts, and thermodynamic parameters (K_ads_ and ΔG^o^_ads_) by using data obtained from WL measurements.

Adsorption isotherm model	Linear form equation	Temp (K)	Slope	Intercept	R^2^	K_ads_ (ppm^−1^)	ΔG^o^_ads_ (kJ/mol)
Freundlich	*log* $\theta$ = $logK$ + *1/n log C*	298	0.14	−0.56	0.95	0.28	−31.55
		308	0.16	−0.71	0.99	0.19	−30.67
		318	0.15	−0.69	0.94	0.20	−30.80
Langmuir	$\frac{c}{\theta }= \frac{1}{\mathrm{K}}+ c$	298	1.12	307.57	1.00	0.00	−20.37
		308	1.20	534.36	1.00	0.00	−18.98
		318	1.32	548.71	1.00	0.00	−18.91
Temkin	$\theta$ = $- \frac{1}{2a}\mathrm{ln}C$ $- \frac{1}{2a}\mathrm{ln}K$	298	9.25	0.45	0.96	1.05	−34.92
		308	8.80	1.66	0.98	1.21	−35.28
		318	10.13	1.29	0.93	1.14	−35.13
Kinetic-thermodynamic	$log\left(\frac{\uptheta }{1-\uptheta }\right)= y logK+y log c$	298	0.59	−1.38	0.97	0.00	−21.09
		308	0.52	−1.39	0.96	0.00	−19.29
		318	0.40	−1.09	0.92	0.00	−18.88

6 $$\frac{C}{\theta }= \frac{1}{{K}_{ads}}+C$$

Where surface coverage is represented by θ, inhibitor concentration is depicted by C while K_ads_ is the adsorption equilibrium constant.

The relationship between the concentrations of *Terminalia bellerica* extract and C/θ is depicted in Fig. 4. The resultant plot is linear, and the adsorption equilibrium constant (K_ads_) can be measured by using the intercept. The corresponding K_ads_ values are 0.0033, 0.0019 and 0.0018 ppm^−1^ at the temperatures 298 K, 308 K, and 318 K, respectively.

The following formula was used to determine the ${\Delta G}_{ads }^{\circ}$ from the K_ads_ values^67^:

7 $$\Delta {G}_{ads }^{{\circ}}=-RTln({10}^{6} \times {K}_{ads})$$

Where ΔG°_ads_ is the  standard free energy of adsorption; R is the gas constant; T is the absolute temperature.

The obtained values of $\Delta {G}_{ads }^{\circ}$, by using Eq. (7), are −20.37, −18.98 and −18.91 kJ mol^−1^ at the temperature of 298 K, 308 K, and 318 K, respectively. In general, values of ΔG^0^_ads_ around −20 kJ/mol or more positive are indicative of the inhibitor's physisorption on the surface, likewise, values of −40 kJ/mol or more negative are indicative of the inhibitor’s chemisorption on the steel surface^68^. Due to possible interaction between the above-mentioned phytochemicals and the surface of the metal, they can be considered as effective corrosion inhibitor. The inhibitor layer prevents corrosion by obstructing the active sites upon the steel's surface and the physical adsorption is predominant.

### Activation parameter

**Figure 5:**
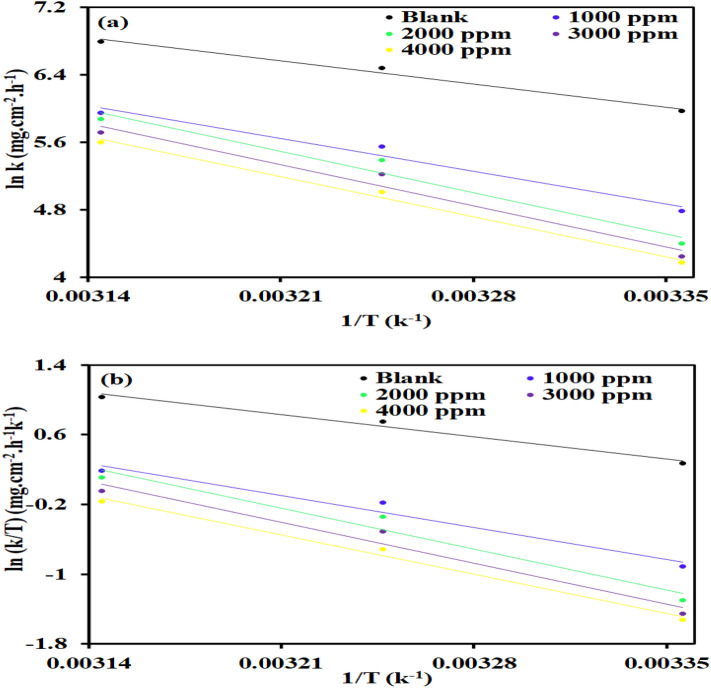
Arrhenius plots and Transition state plots for steel dissolution with and without different concentrations of Terminalia bellerica in 1.0 M H_2_SO_4_ solution**.**

Figure 5a demonstrates a linear relationship between ln (CR) and 1/T, from which E_a_ can be figured out. Using Eq. (8), E_a_ can be calculated and with accordance to the Arrhenius law, E_a_ varies as temperature rises, which accelerates metal corrosion.

8 $${E}_{a}= -Slope\times 8.314$$

When an inhibitor was added, E_a_ (Table 6) was increased from 32.69 to 58.19 kJ/mol, demonstrating the physical adsorption of the inhibitor onto the metal surface^69^. Due to the development of a protective layer over the exposed steel surface, activation energy has therefore increased, suggesting that dissolved steel has been reduced.

**Table 6:** Activation parameters of dissolution reaction of steel in 1 M H_2_SO_4_ with at different concentrations of *Terminalia bellerica.*

Inhibitor	Conc. of inhibitor (ppm)	E_a_ (kJ/mol)	∆H* (kJ/mol)	∆S* (J mol/K)
Blank	0.00	32.69	29.50	−93.96
*Terminalia bellerica*	1000	46.09	42.62	−58.59
	2000	58.19	54.46	−21.02
	3000	58.12	54.40	−22.58
	4000	56.28	52.60	−29.67

### Adsorption parameter for entropy and enthalpy

The following equation has been used to evaluate the entropy and enthalpy of adsorption:

9 $$ln\left\{\frac{CR}{T}\right\}=log \left\{\frac{R}{{N}_{a}h}\right\}+ \frac{{\Delta S}_{a}}{R }- \frac{{\Delta H}_{a}}{RT}$$

In this formula, N_a_, h, ∆H_a,_ and ∆S_a_ are Avogadro's number, Planck constant, standard activation enthalpy and standard activation entropy, respectively.

By using graph ln (CR/T) versus /T, as shown in Fig. 5b, the ∆H_a_ and ∆S_a_ parameters have been determined and illustrated in Table 6. It can be inferred, the metal is very well protected because a corrosion-preventing energy barrier is maintained, as demonstrated by the fact that H_a_ is greater when there an inhibitor used (54.46 kJ/mol) compared to solution without inhibitor (29.50 kJ/mol). The inhibitor adsorption leads to a rise in the corrosion reaction’s enthalpy (+ ve, endothermic). The introduction of the inhibitor produced an entropy value of -21.02 J/mol/K as opposed to the blank solution having entropy of −93.96 J/mol/K.

### Electrochemical studies

#### Potentiodynamic polarization (PDP) study

**Figure 6:**
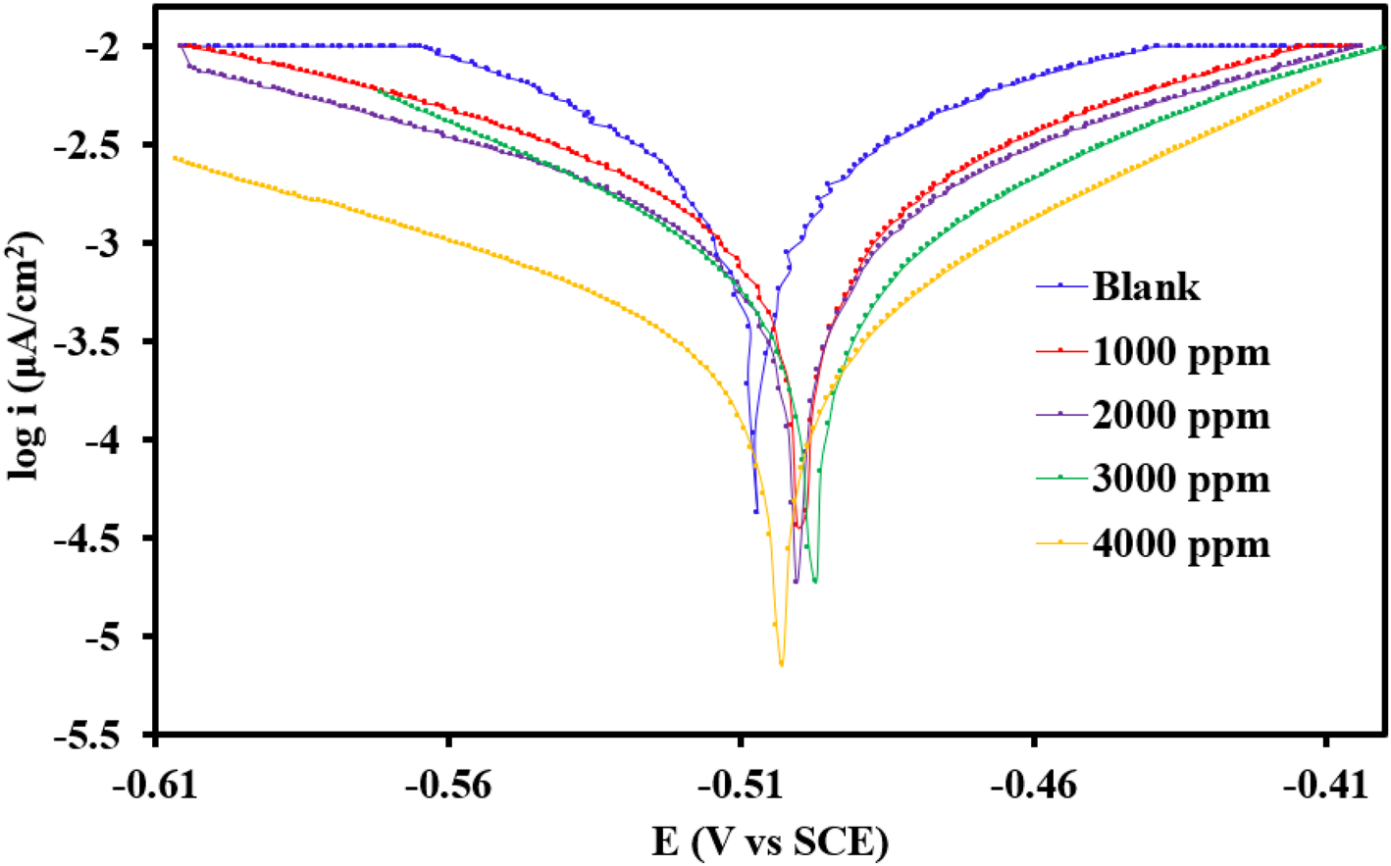
Tafel polarization curves in 1 M H_2_SO_4_ without and with various concentrations of *Terminalia bellerica* extract.

In the corrosive solvent with various extract concentrations, Fig. 6 clearly demonstrates the anodic and cathodic polarization responses of steel coupons, while Table 7 enlists the related corrosion outcomes and inhibition efficiencies.

**Table 7:** Potentiodynamic polarization parameters for steel dissolution without and with various concentrations of extract.

Inhibitor concentration (mg/L)	*E*_corr_ (V vs. SCE)	*I*_corr_ (A cm^-2^)	*β*_a_ (V/dec)	*β*_c_ (V/dec)	CR (mpy)	Efficiency (*η* %)
0	−0.506	2.9 × 10^–3^	0.09527	0.06263	34.03	0
1000	−0.480	1.2 × 10^–3^	0.1093	0.1204	14.52	58.62
2000	−0.598	1.0 × 10^–3^	0.0407	0.03828	12.15	65.51
3000	−0.427	3.4 × 10^–4^	0.04677	0.03600	04.23	88.27
4000	−0.523	2.6 × 10^–4^	0.07052	0.10421	02.92	91.03

According to the Tafel curves, the anodic and cathodic section current density is decreasing in the presence of the *Terminalia bellerica* extract. This behavior demonstrate that the inhibitor has the ability to suppress the cathodic hydrogen evolution as well as anodic metal oxidation^70^. The anodic as well as cathodic Tafel graphs (βa and βc) with the *Terminalia bellerica* extract has been changed with certain concentrations which show that the inhibitor affects the cathode hydrogen gas evolution and also interfering with the anode iron dissolving process^71^. Thus, in a solution of 1 M H_2_SO_4_, the *Terminalia bellerica* extract regulates both cathodic and anodic corrosion reactions^72,73^. This should indicate that adding more *Terminalia bellerica* extract prevents the steel corrosion process. According to Table 7, the corrosion current density reduces as *Terminalia bellerica* extract concentration increases^74^. At a concentration of 4000 mg/L, the lowest corrosion current density is 2.610^–4^ A/cm^2^ and the best inhibition efficiency showed 91.03%. In addition, Table 7 demonstrates a decline in the corrosion rate in the presence of extract; all this is due to the adsorption capacity of the extract on the surface of the metal which provides remarkable resistance against metal dissolution.

### Electrochemical impedance spectroscopy

**Figure 7:**
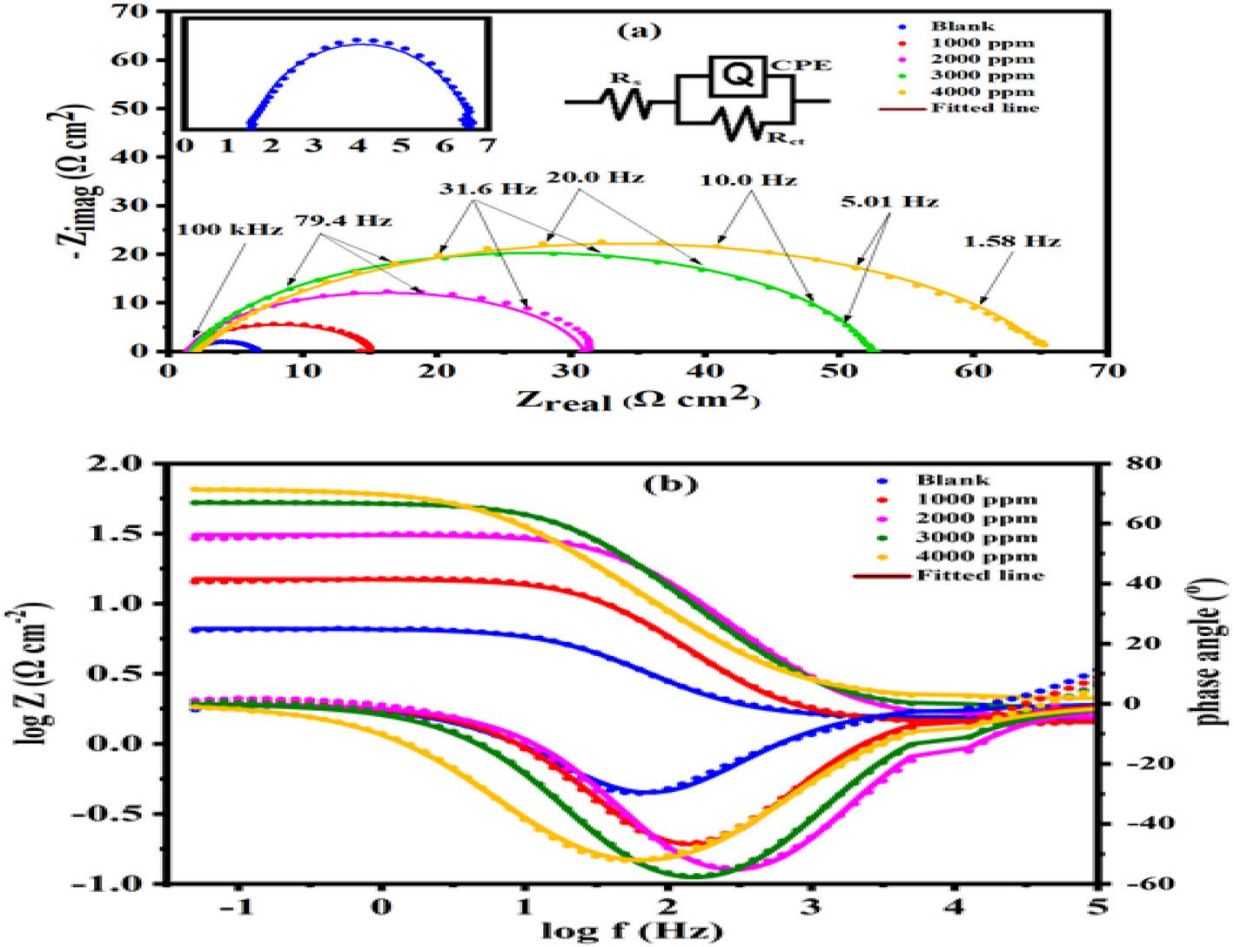
Nyquist (**a**) and Bode plots (**b**), and equivalent circuit of constant phase element (CPE) for steel in 1 M H_2_SO_4_ without and with various concentrations of *Terminalia bellerica* extract at 298 K.

The resulting Nyquist as well as Bode diagrams are displayed in Fig. 7a, b, and resulted findings are shown in Table 8. Figure 7a also, shows the circuit which was employed in this study.

**Table 8:** Electrochemical parameters calculated from EIS measurements on mild steel electrode in 1.0 M H_2_SO_4_ solutions without and with various concentrations of *Terminalia bellerica* at 298 K.

Inhibitor	Conc (ppm)	R_s_ (Ω cm^2^)	R_ct_ (Ω cm^2^)	Y_o_ (μ/Ω s^n^/cm^2^)	n	C_dl_ (μF/cm^2^)	Chi squared (χ^2^)	S	α°	θ	η_z_ (%)
Blank	–	1.533	5.109	1500.00	0.822	522.366	2.56 × 10^–3^	−0.308	−29.76	–	–
*Terminalia bellerica*	1000	1.423	13.55	340.90	0.8741	157.125	2.21 × 10^–3^	−0.449	−46.52	0.623	62.30
	2000	1.531	29.46	116.30	0.8772	52.538	2.47 × 10^–3^	−0.421	−54.60	0.827	82.66
	3000	1.803	50.69	157.30	0.8618	72.483	1.21 × 10^–3^	−0.561	−57.40	0.899	89.92
	4000	2.092	63.45	473.40	0.7789	175.023	9.56 × 10^–4^	−0.573	−52.17	0.919	91.95

*R*_*s*_ solution resistance, *R*_*ct*_ charge transfer resistant, *Y*_*0*_*, n* constants of phase elements, *C*_*dl*_ double layer capacitance, *S* slopes of the Bode impedance magnitude at intermediate frequencies, *α°* maximum phase angle values, *θ* surface coverage, *η*_*z*_ inhibition efficiency.

From Fig. 7a, the diameter of the capacitive loops increased when the extract was added compared to that one without extract, indicating that this additive significantly slowed down the dissolution of steel^75,76^. The profiles of the spectra seem similar, and the semicircular form suggests that the corrosion mechanism was not affected by the presence or absence of the plant extract^77^. The outcomes of Table 8 indicate that increasing the inhibitor concentration while it reaches the highest efficiency at 4000 ppm increases the value of charge transfer resistance and inhibition efficiency. According to the Bode angle diagram, just after introduction of the inhibitor, the establishment of a protective layer on the surface, causes the curves to widen while moving to the left (toward smaller frequencies). In this study, R_ct_ values increase with rise in inhibitor concentrations, which indicates that the extract from *Terminalia bellerica* is easily absorbable on the steel's surface, with a highest efficiency of 91.95% at a concentration of 4000 mg/L being observed. A presence of non-ideal capacitor can be indicated by the slightly flattened semi-circles that result from the electrode flaws and/or surface reactions^78^. In an analogous circuit, the capacitive element's variations are represented by a constant phase element (CPE). The variation of the capacitor behavior for a CPE is represented by a value of 0 < n < 1 (n = 1 represents a pure capacitor)^72,73^. For n, a value of around 0.8 was found in this study as shown in Table 8.

### Analysis of phytochemicals

The phytochemicals found in the *Terminalia bellerica* extract are further described in Table 9.

**Table 9:** Phytochemical analysis of *Terminalia bellerica* extract.

S. no	Phytochemicals	Test	Result
1	Alkaloids	Wagner’s test	_ _ _ _
		Mayer’s Test	
2	Flavanoids	Conc. hydrochloric acid test	+ +
3	Saponins	Froth test	_ _
4	Quinones	Concentrated sulphuric acid test	+ +
5	Coumarins	Alcoholic NaOH test	_ _
6	Sugar	Fehling solution test	+ +

### Test for alkaloids

#### Wagner’s test

The extract was treated with Wagner's reagent. The development of a brownish-reddish precipitate hints the presence of alkaloids^79^.

#### Mayer’s test

Solution was tested for alkaloids using Mayer's reagent. Presence of alkaloids revealed by yellow color in the solution^79^.

#### Test for flavonoids

After addition of several drops of concentrated HCl to a small amount of the plant extract solution the quick appearance of red color was used to identify the flavonoids^80^.

#### Test for saponins

Five milliliters of water and five milliliters of filtrate are mixed vigorously. The development of steady foam is a clue that saponins are present.^81^.

#### Test for quinones

Upon addition of one mL of the extract in H_2_SO_4_, a color change is observed accordingly the existence of quinones is verified^82^.

#### Test for coumarin

To about 2 ml of the extract, a few drops of an alcoholic NaOH solution were added. The appearance of a yellow color is an evidence that coumarin exists^82^.

### Test for sugars

#### Fehling solution test for sugars

Both the Fehling solutions A and B were combined in a one-to-one ratio and then boiled for 1 min. About 1 ml of an extract was taken from that mixture and then heated for 5 to 10 min in the water bath. When yellowish or brick red precipitates first appeared, The presence of carbohydrates is confirmed^79,80^.

#### UV visible spectroscopy

**Figure 8:**
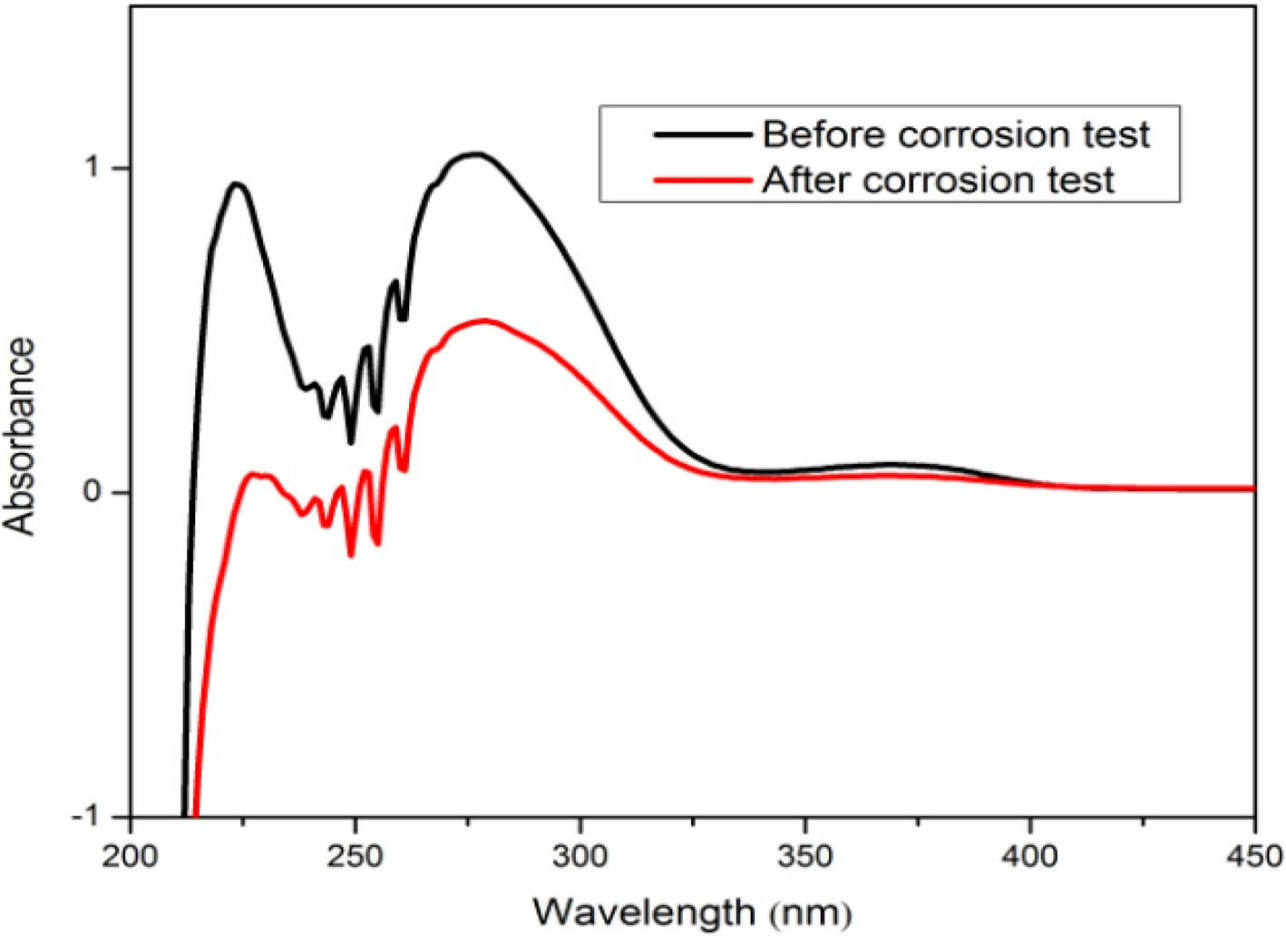
UV spectrum of *Terminalia bellerica* extract before and after the corrosion inhibition performance.

UV spectra of *Terminalia bellerica* extracts were analyzed before and after corrosion. The acidic medium having an inhibitor, while the steel was not yet submerged, showed a significantly high absorbance, as shown by the spectra. This was compared to that of the abrasive medium in which the steel was submerged for 24 h, as shown in Fig. 8. When the steel specimen was soaked in the corrosive media containing an inhibitor, a complex between the surface of the steel and the inhibitor was formed. It is clear that the various phytochemicals of the inhibitor have formed a protective layer after being adsorbed to the metal^83^.

### Surface investigation

#### Scanning electron microscope (SEM)

**Figure 9:**
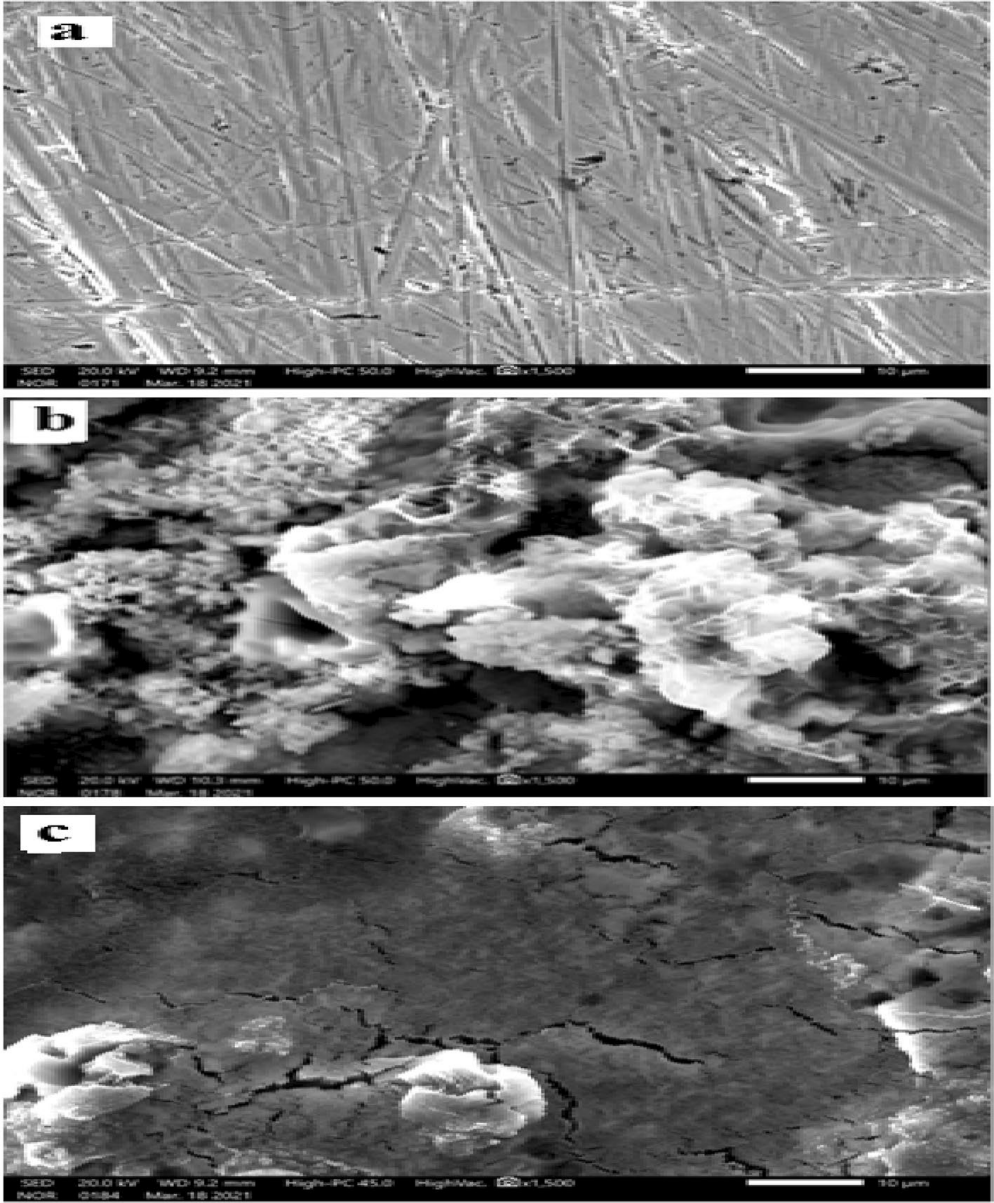
Surface pictures of (**a**) cleaned mild steel (**b**) corroded in 1 M H_2_SO_4_, and (**c**) inhibited by 4000 mg/L *Terminalia bellerica*’s fruit extract.

The morphological changes to the cleaned steel surfaces after a 24-h immersion in 1M H_2_SO_4_ with or without extract are illustrated in Fig. 9. It can be observed from Fig. 9a that the polished metal surface was not homogeneous and had a few flaws that might serve as corrosion points. Figure 9b shows a very rough surface caused by the rapid and powerful corrosion processes that took place during the immersion of steel in blank H_2_SO_4_. Accordingly a protective layer on the steel's surface was created by adding an inhibitor to 1 M H_2_SO_4_, thereby reducing the extent of surface damage caused by corrosion (see Fig. 9c)^84^.

### Analysis by atomic force microscope (AFM)

**Figure 10:**
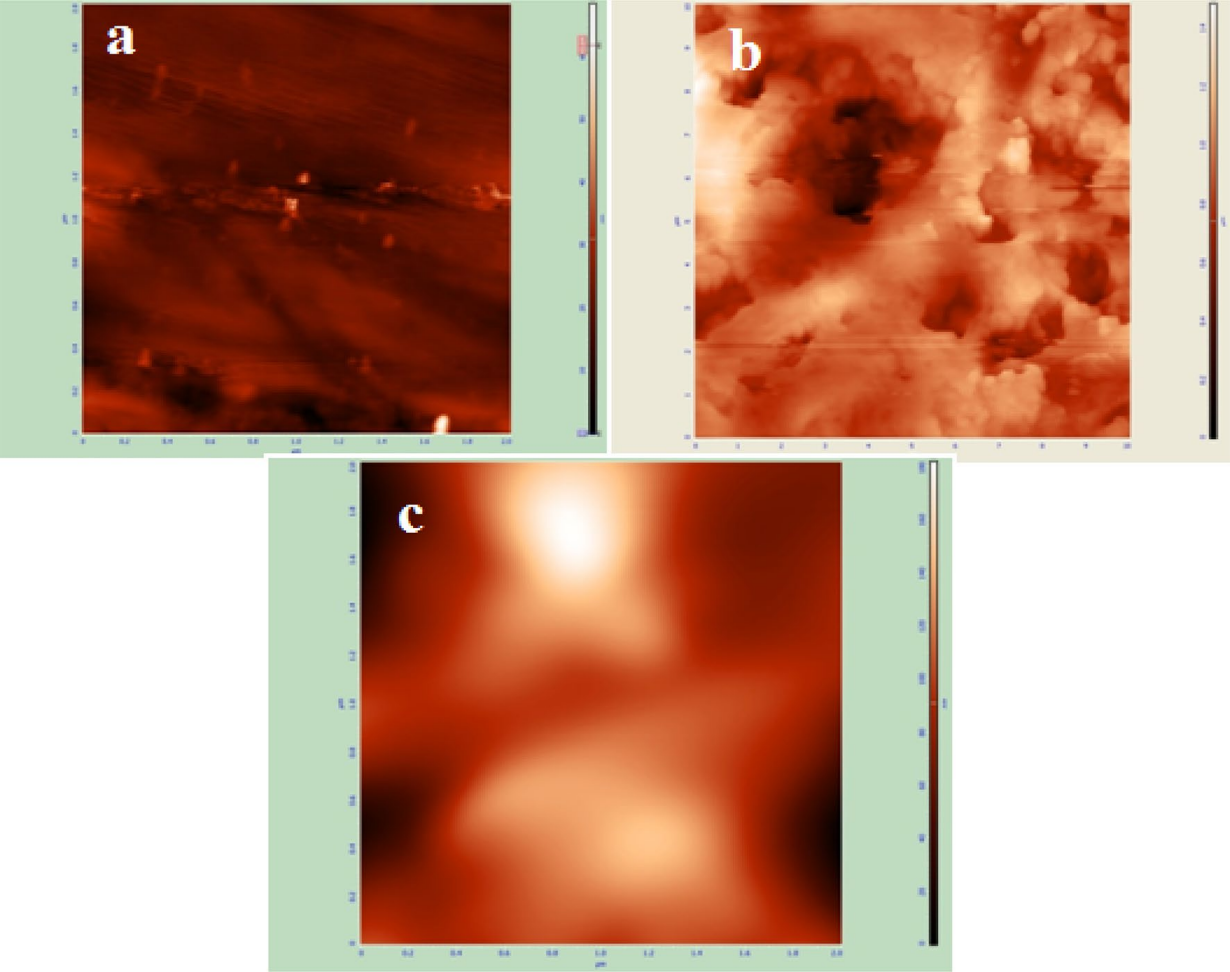
AFM images of (**a**) cleaned mild steel (**b**) corroded in 1 M H_2_SO_4_, and (**c**) inhibited by 4000 mg/L *Terminalia bellerica*’s fruit extract.

Figure 10a–c described the two-dimensional AFM pictures of polished, uninhibited, and protected steel in 1 M H_2_SO_4_ with *Terminalia bellerica* extract, respectively. For polished steel, the surface roughness value is 2.08 nm. The upper surface of the steel was extensively corroded in the absence of *Terminalia bellerica* extract because steel was dissolved in the acidic solution and the average surface roughness in this condition was 145.75 nm. The surface roughness measured in the presence of *Terminalia bellerica* extract was 23.27 nm. From the values of roughens, it is evident that a protective layer was developed on the metal's surface^85^.

### Molecular reactivity

#### Global reactivity

**Figure 11:**
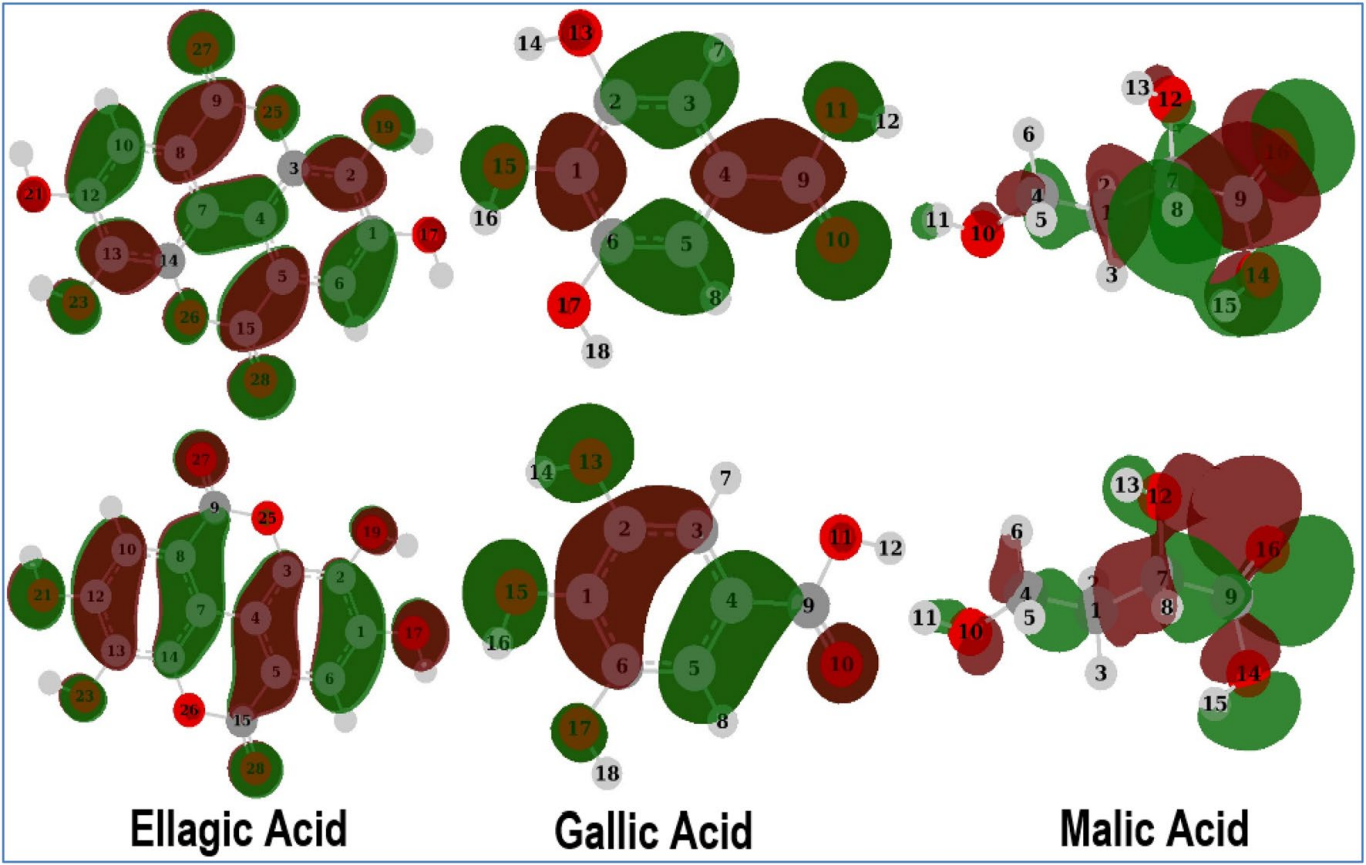
Frontier molecular orbitals: LUMOs (upper row) and HOMOs (lower row).

Organic molecules usually show good reactivity toward metallic surfaces or metal ions because they are rich in π-electrons and lone pairs of hetero atoms^86^. Such loosely tight electrons with low ionization potentials are easily reallocated in the vicinity of d-orbitals of common transition metals^87,88^. This donating behavior of organic molecules can be evaluated by calculating the global reactivity descriptors including ionization potential, electronegativity, chemical harness, electron affinity etc. (Table 10) in addition to the band gap between the HOMO and LUMO (Fig. 11). However, the B3LYP method underestimates the HOMO eigen value by average absolute error of 3.10 eV according to Zhang and Musgrave et. al.^55^.

**Table 10:** Calculated electronic reactivity indices for ellagic, gallic and malic acids.

Molecular parameters^a^		Ellagic		Gallic		Malic	
		B3LYP	TD-B3LYP^b^	B3LYP	TD-B3LYP^b^	B3LYP	TD-B3LYP^b^
E_LUMO_	–	−1.847	−3.91	−1.056	−3.158	−0.232	−2.657
E_HOMO_	–	−6.086	−8.723	−5.986	−8.603	−7.333	−10.219
ΔE (E_LUMO_ – E_HOMO_)	(E_LUMO_ – E_HOMO_)	4.239	4.813	4.930	5.444	7.101	7.562
Ionization potential (IP)	– E_HOMO_	6.086	8.723	5.986	8.603	7.333	10.219
Electron affinity (EA)	– E_LUMO_	1.847	3.91	1.056	3.158	0.232	2.657
Electronegativity (χ)	(IP + EA)/2	3.967	6.317	3.521	5.881	3.55	6.438
Chemical potential (μ)	−χ	−3.967	−6.317	−3.521	−5.881	−3.55	−6.438
Chemical hardness (η)	(IP-EA)/2	2.12	2.407	2.465	2.723	3.782	3.781
Chemical softness (σ)	1/ η	0.472	0.415	0.406	0.367	0.264	0.264
Global electrophilicity (ω)	χ^2^/2η	3.712	8.289	2.515	6.351	1.666	5.417
ΔN	(χ_metal_ − χ_inh_) **/**2(η_metal_ + η_inh_)	0.500	0.288	0.508	0.308	0.418	0.228

^a^All parameters in eV except ΔN is dimensionless quantity.

^b^All values are corrected according to the procedure in Ref.^55^.

Although it cannot be predicted about the LUMO eigen value that agrees with the recorded electron affinities; therefore, a calculated accurate band gap is a challenge. Nevertheless, the estimated band gap using time-dependent (TD) B3LYP is fairly accurate. Therefore, a correction to the eigen value of HOMO could be used with the TD band gap for accurate estimation of LUMO and hence an accurate prediction for the reactivity descriptors. Correction of HOMO eigen value is carried out using the formula: $-{HOMO}_{corr}=A+B(-{HOMO}_{cal})$ where A = 1.42 and B = 1.20; correlation parameters of B3LYP functional^55^. Then, an accurate value of LUMO is obtained using the formula: $LUMO=HOMO+{\Delta E}_{gap}$. The band gap ${\Delta E}_{gap}$ calculated by TD-B3LYP for Ellagic, Gallic and Malic acids are 4.813, 5.444 and 7.562 eV, respectively (Table 10 and Fig. 11). Because the HOMO and LUMO wave functions of a moderate-sized band gap are easily combined, the molecule is quickly polarized and requires less energy to be excited^56,89^. Therefore, the calculated low energy gaps promote the reactivity of these molecules toward the metal surface and hence their ability as corrosion inhibition candidates in the following order Ellagic > Gallic > Malic acid.

The calculated eigenvalues for HOMO of Ellagic, Gallic, and Malic acid molecules are −8.723, −8.603, and −10.219 eV, respectively (Table 10). These low orbitals eigen values indicate that the electrons occupying these orbitals need few electron volts of energy to be ionized (i.e.: + 8.723, + 8.603, and + 10.219 eV for Ellagic, Gallic, and Malic acid, respectively) and hence can be easily shared into the metal surface. As expected, the HOMOs are mainly localized on the π-system of the carbonyl group and phenyl ring in both Ellagic and Gallic acids and on the carbonyl group and bonds including covalently attached oxygen atoms (Fig. 11). Moreover, the chemical hardness for the studied molecules was estimated as suggested by R. G. Pearson in “chemical hardness”^56^. Pearson suggested that in molecules where the HOMO is filled with electrons, the electronegativity divides the band gap into two parts and the addition of these parts gives the chemical hardness. Having low chemical hardness 2.407, 2.723, and 3.781 (Table 10) for Ellagic, Gallic, and Malic acid, respectively indicates that they are good candidates for strong and efficient interactions with the metal surface. Furthermore, the mutual tendency of charge transfer between two interacting entities, in this case, inhibitor and metal surface, could be calculated as follows^90^:

10 $$\Delta N=\frac{({\chi }_{metal}-{\chi }_{inh.})}{2({\eta }_{metal}+{\eta }_{inhi.})}$$

where $\chi$ and $\eta$ are electronegativity and chemical hardness, respectively. The predicted ΔN for the Ellagic, Gallic, and Malic acid are 0.288, 0.308, and 0.228, respectively (Table 10) revealing the good tendency of these molecules to mutually share electrons with the metal surface. Overall, global reactivity indices suggest a feasible mutual inhibitor-metal interactions between Ellagic, Gallic, and Malic acid and mild steel nominating them for effective corrosion suppression and explaining their recorded electrochemical high inhibition efficiency.

### Natural bond orbital analysis

The results of NBO analysis are summarized in Tables 11, 12 and 13 for molecules of Ellagic, Gallic, and Malic acid, respectively.

**Table 11:** Calculated NBOs of ellagic acid at expected adsorption sites.

Type*	Occupancy	Energy	NBO	s % (atom 1)	p % (atom 1)	s % (atom 1)	p % (atom 2)
LP(2)O_27_	1.82835	−0.25629	p^1.00^	0.11	99.62	–	–
LP(2)O_28_	1.82835	−0.25629	p^1.00^	0.11	99.62	–	–
BD(2)C_7_-C_8_	1.63250	−0.27030	0.6930 p + 0.7209 p	0.00	99.98	0.00	99.98
BD(2)C_4_-C_5_	1.63251	−0.27030	0.6930 p + 0.7209 p	0.00	99.98	0.00	99.98
BD(2)C_10_-C_12_	1.69627	−0.28469	0.7068 p + 0.7074 p	0.00	99.95	0.00	99.96
BD(2)C_1_-C_6_	1.69628	−0.28470	0.7074 p + 0.7068 p	0.00	99.96	0.00	99.95
BD(2)C_13_-C_14_	1.63195	−0.28731	0.6970 p + 0.7170 p	0.00	99.95	0.00	99.96
BD(2)C_2_-C_3_	1.63196	−0.28732	0.6970 p + 0.7170 p	0.00	99.95	0.00	99.96
LP(2)O_23_	1.84729	−0.32988	p^1.00^	0.00	99.87	–	−
LP(2)O_19_	1.84729	−0.32988	p^1.00^	0.00	99.87	−	−
LP(2)O_26_	1.75054	−0.33587	p^1.00^	0.00	99.88	−	−
LP(2)O_25_	1.75054	−0.33587	p^1.00^	0.00	99.88	−	−
LP(2)O_21_	1.88602	−0.33587	p^1.00^	0.00	99.90	−	−
LP(2)O_17_	1.88602	−0.33587	p^1.00^	0.00	99.90	−	−
BD(2)C_9_-O_27_	1.98285	−0.38115	0.5620 p + 0.8271 p	0.00	99.82	0.00	99.66
BD(2)C_15_-O_28_	1.98285	−0.38115	0.5620 p + 0.8271 p	0.00	99.82	0.00	99.66

*LP(1): refers to first lone pair, LP(2): second lone pair, etc. BD(1): bonding orbital of a single bond, BD(2): for double bond.

**Table 12:** Calculated NBOs of gallic acid at expected adsorption sites.

Type*	Occupancy	Energy	NBO	s % (atom 1)	p % (atom 1)	s% (atom 1)	p % (atom 2)
BD(2)C_3_-C_4_	1.68327	−0.24870	0.6958 p + 0.7182 p	0.00	99.96	–	99.98
LP(2)O_10_	1.84659	−0.25446	p^1.00^	0.00	99.75	–	–
BD(2)C_5_-C_6_	1.69592	−0.26659	0.7122 p + 0.7020 p	0.00	99.95	–	99.96
BD(2)C_1_-C_2_	1.62619	−0.26871	0.7207 p + 0.6933 p	0.00	99.96	–	99.95
LP(2)O_13_	1.86908	−0.31224	p^1.00^	0.00	99.88	–	–
LP(2)O_11_	1.82499	−0.32605	p^1.00^	0.00	99.88	–	–
LP(2)O_15_	1.87948	−0.33081	p^1.00^	0.00	99.90	–	–
LP(2)O_17_	1.89289	−0.33607	p^1.00^	0.00	99.91	–	–
BD(2)C_9_-O_10_	1.98484	−0.37258	0.5477 p + 0.8367 p	0.00	99.80	–	99.67
BD(1)C_3_-H_7_	1.97675	−0.51611	0.7970 sp^2.26^ + 0.6039 s	30.66	69.31	99.95	0.00

*LP(1): refers to first lone pair, LP(2): second lone pair, etc. BD(1): bonding orbital of a single bond, BD(2): for double bond.

**Table 13:** Calculated NBOs of Malic acid at expected adsorption sites.

Type*	Occupancy	Energy	NBO	s % (atom 1)	p % (atom 1)	s% (atom 1)	p % (atom 2)
LP(2)O_16_	1.82816	−0.24696	p^1.00^	0.01	99.70	–	–
LP(2)O_10_	1.95998	−0.30223	p^1.00^	0.12	99.79	–	–
LP(2)O_12_	1.95228	−0.30692	p^1.00^	0.46	99.43	–	–
LP(2)O_14_	1.83031	−0.33199	p^1.00^	0.01	99.86	–	–
BD(2)C_9_-O_16_	1.99182	−0.38099	0.5689 p + 0.8224 p	0.11	99.71	0.17	99.47
BD(1)C_1_-H_2_	1.97871	−0.50412	0.7960 p + 0.6053 s	23.67	76.28	99.95	0.00

*LP(1): refers to first lone pair, LP(2): second lone pair, etc. BD(1): bonding orbital of a single bond, BD(2): for double bond.

The Tables list natural bond orbitals at expected inhibitor-metal interactions in descending order according to their energies along with their type of bonding, electron occupancy, energy, hybridization, and percent of s and p characters. Moreover, the surface densities of these orbitals are plotted in Table S1 to Table S3 with the same descending order. Analyzing NBOs allows us to determine the importance of individual sites of a molecule to come into interaction with the steel surface. In other words, NBOs order is an order of donating ability of the electron density involved in an interaction. The results in Table 11 show that the electron density of Ellagic acid molecule can be shared into the metal surface in the following order: LP(2)O_27_ > LP(2)O_28_ > BD(2)C_7_-C_8_ > BD(2)C_4_-C_5_ > BD(2)C_10_-C_12_ > BD(2)C_1_-C_6_ > BD(2)C_13_-C_14_ > BD(2)C_2_-C_3_ > LP(2)O_23_ > LP(2)O_19_ > LP(2)O_26_ > LP(2)O_25_ > LP(2)O_21_ > LP(2)O_17_ > BD(2)C_9_-O_27_ > BD(2)C_15_-O_28_. The plots in (Table S1 to S3) show the source orbitals for these NBOs; for example, bonding orbital LP(2)O_27_ originates from HOMO of the Ellagic acid molecule. In other words, the HOMO of the Ellagic acid molecule is localized on the lone pair of the O_27_ atom and it is easier to interact with the steel surface than the other NBOs. In this order, the electronic density of BD (2)C_15_-O_28_ orbital of the double bond C_15_-O_28_ comes late at HOMO-15 which indicates a weaker ability to reallocate its density into *d*-orbitals of the steel metal. In accordance, the donating ability of Gallic acid molecule shows the following order: BD(2)C_3_-C_4_ > LP(2)O_10_ > BD(2)C_5_-C_6_ > BD(2)C_1_-C_2_ > LP(2)O_13_ > LP(2)O_11_ > LP(2)O_15_ > LP(2)O_17_ > BD(2)C_9_-O_10_ > BD(1)C_3_-H_7_ as listed in Table 12 while corresponding orbital densities from HOMO to HOMO-9 are listed in Table S2. Moreover, NBOs of Malic acid show donating behavior following the order: LP(2)O_16_ > LP(2)O_10_ > LP(2)O_12_ > LP(2)O_14_ > BD(2)C_9_-O_16_ > BD(1)C_1_-H_2_. It is worth noticing that the number of donating sites follows the order Ellagic > Gallic > Malic which in turn favors the adhering of metal surface in the same order.

### Local reactivity

**Figure 12:**
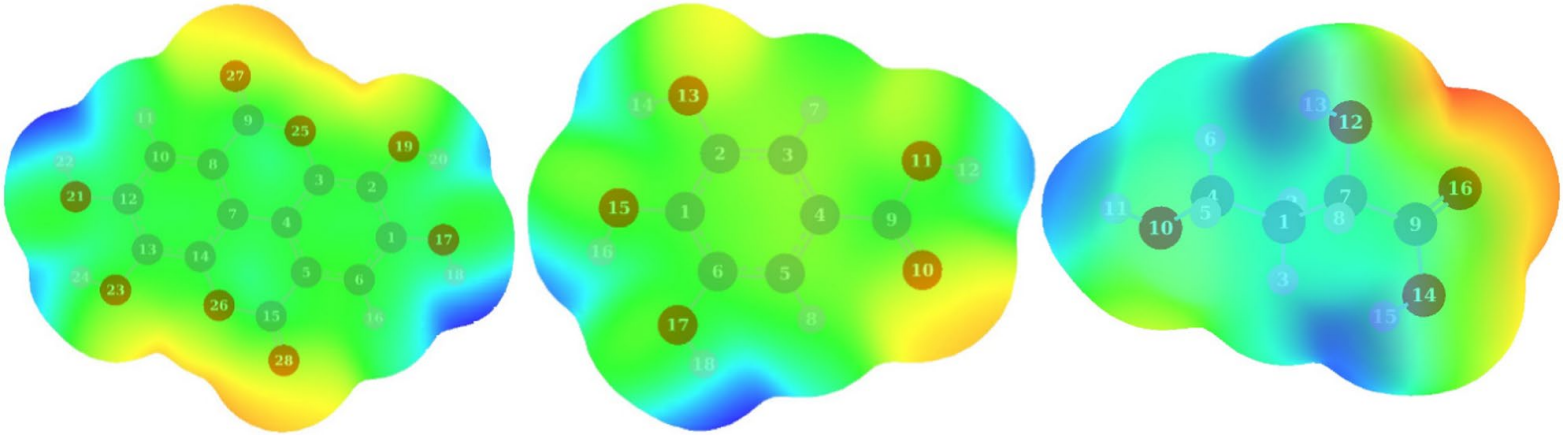
Molecular electrostatic potential (MEP) map: from left (ellagic, gallic and malic acids).

While global reactivity explores the tendency of a molecular entity to be involved in chemical change, local reactivity figures out the sites within this entity to react with another one. For a molecule contacting metal surface and suppress corrosion reaction, it is worthy to have electron rich regions more than electron-poor ones. These regions could be explored using a calculated molecular electrostatic potential (MEP) map shown in Fig. 12.

In such map, electron-rich sites having low electrostatic potential are designated with red color and electron-poor sites are blue colored while sites with green color indicate moderate charge density content. The mapped surfaces presented in Fig. 12 show that the distribution of the colors follows the order: green > blue > red. The existence of wide areas with moderate electron density is common in organic molecules; however, maps of Ellagic, Gallic, and Malic molecules are characterized by electron-rich regions much more than electron poor-regions. The red regions are localized around carbonyl groups which agree with the order of the NBOs where HOMOs are corresponding to the lone pairs of oxygen atoms of the carbonyl groups suggesting that these groups have the priority to contact the metal surface.

Although the localization of electronic density over a particular site indicates its tendency to contact metal surface, which is useful, the magnitude of this conduct cannot be estimated from the MEP map. The magnitude of a particular site to be either nucleophile or electrophile could be determined using Fukui functions. In this procedure, the neutral molecule is charged by either a negative or a positive charge than the second derivative of energy is probed, for each atom, with respect to number of electrons and the external potential owing while molecules approaching each other as follows:

11 $$f\left(r\right)={\partial }^{2}E/\partial N \partial \nu$$

A good approximation to solve this equation is to use “condensed” Fukui functions on an atom-by-atom basis^91^:

12 $${f}_{k}^{-}=[{q}_{k}\left(N\right)-{q}_{k}\left(N-1\right)]$$

13 $${f}_{k}^{+}=[{q}_{k}\left(N+1\right)-{q}_{k}\left(N\right)]$$

Where ${f}_{k}^{-}$ and ${f}_{k}^{+}$ are electrophilic and nucleophilic Fukui indices, respectively and ${q}_{k}$ is the atomic charge. We used Hirshfeld charges since it is corrected to bond orders and is much accurate for molecules having single-double bonds in conjugations. We listed the predicted Fukui indices for the Ellagic, Gallic, and Malic molecules in Table 14 according to the descending order of electrophilic Fukui function since it is of more interest for inhibitor-metal interactions.

**Table 14:** Calculated condensed Fukui functions for ellagic, gallic and malic acid molecules.

Ellagic			Gallic			Malic		
Atoms	$f^{ + }$	$f^{ - }$	Atoms	$f^{ + }$	$f^{ - }$	Atoms	$f^{ + }$	$f^{ - }$
C_3_	0.018	0.015	O_17_	0.430	0.032	C_1_	0.027	0.017
C_14_	0.018	0.015	O_13_	0.118	0.034	O_12_	0.089	0.027
O_25_	0.023	0.023	C_6_	0.044	0.042	C_4_	0.048	0.030
O_26_	0.023	0.023	C_2_	0.077	0.044	C_7_	0.042	0.038
C_4_	0.032	0.023	O_15_	0.116	0.054	O_10_	0.152	0.043
C_7_	0.032	0.023	O_11_	0.036	0.057	O_14_	0.093	0.080
O_17_	0.07	0.024	C_5_	0.067	0.062	C_9_	0.069	0.157
O_21_	0.07	0.024	C_4_	0.084	0.067	O_16_	0.226	0.162
C_1_	0.061	0.032	C_3_	0.040	0.070			
C_12_	0.061	0.032	C_1_	0.098	0.084			
O_19_	0.05	0.035	C_9_	0.018	0.117			
O_23_	0.05	0.035	O_10_	0.076	0.122			
C_6_	0.041	0.043						
C_10_	0.041	0.043						
C_5_	0.04	0.044						
C_8_	0.04	0.044						
C_2_	0.028	0.054						
C_13_	0.028	0.054						
C_9_	0.015	0.055						
C_15_	0.015	0.055						
O_27_	0.068	0.073						
O_28_	0.068	0.073						

Calculated using Hirshfeld charges at B3LYP/6-31G(d,p).

Some good information can be inferred from Table 14, for example, in Ellagic acid, the oxygen atoms of carbonyl groups carry the highest $({f}_{k}^{-}=0.073)$ electrophilic Fukui indices (O_28_ and O_27_) while those of hydroxyl groups carry lower indices ranging between 0.024 and 0.035 and those involved in the carbon skeleton are of the lowest electrophilic sites (Table 14). The same trend applies to oxygen atoms of Gallic acid; however, the presence of conjugation within the carbon rings provides stronger electrophilic sites than those of oxygen atoms. Finally, the Malic acid has the lowest electrophilic Fukui indices, though its carbonyl is much electrophilic than the corresponding carbonyl of Gallic acid which could be attributed to the conjugation of the latter with the phenyl ring.

### Monte Carlo simulations

**Figure 13:**
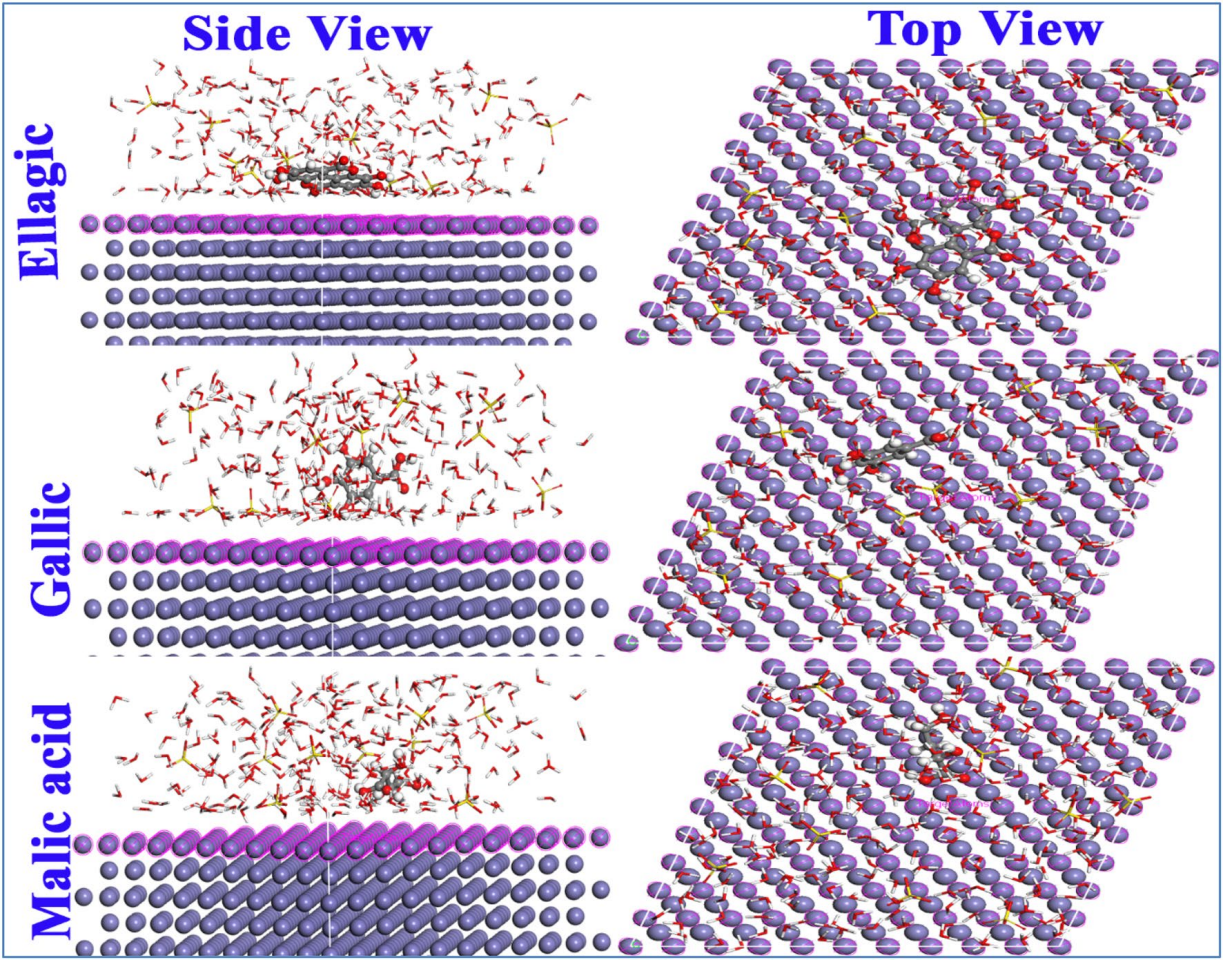
Side and top views of the most appropriate configuration for adsorption of neutral molecules on Fe (110) Surface obtained by MC simulations in the aqueous solution.

The modeling at the molecular level was done for the adsorption of *Terminalia bellerica* Fruit Extract (Ellagic, Gallic, and Malic acid) on steel (or Fe (1 1 0)) in the gas phase and the aqueous phase. Figure 13 displays the most stable configurations of the adsorbed *Terminalia bellerica* Fruit Extract modeled using MC in the aqueous phase settings. Figure 13 shows the close contact between *Terminalia bellerica* Fruit Extract constituents and the steel surface. This confirms their ability to compete with water and the corrosive ions in adsorption and so enhance protection^92^. It is noteworthy findings of the MC support the DFT investigation, which showed that the Ellagic structure has a high reactivity (completely flat and parallel adsorption) compared to Gallic and Malic acid, which are partially flat adsorbed. This may be used to support the inhibitory performance of the studied structures. Table 15 displays the binding energy and the different forms of the adsorption energy values for the Ellagic, Gallic, and Malic acid molecules. The spontaneous tendencies of *Terminalia bellerica* Fruit Extract constituents for adsorption are confirmed by negative adsorption energies values^93^. This is due to the numerous active adsorption sites found on the extract molecules. Based on the binding energy values, Ellagic has the highest binding energy followed by Gallic and Malic acid. This arrangement supports the outcomes of the DFT computation.

**Table 15:** The outputs and descriptors calculated by the Monte Carlo simulation for adsorption of ellagic, gallic and malic acid molecules on Fe (110).

Descriptors*	Gas phase			Aqueous phase		
	Ellagic	Gallic	Malic	Ellagic	Gallic	Malic
Total energy	−21.850	−109.310	−62.708	−7765.7	−7794.0	−7626.8
Adsorption energy	−153.146	−88.060	−84.587	−7953.5	−7938.7	−7924.2
Rigid adsorption energy	−146.755	−84.648	−63.882	−8006.1	−7993.3	−7961.6
Deformation energy	−6.390	−3.412	−20.705	52.5	54.5	37.5
(dE_ads/dNi_)	−153.146	−88.061	−84.587	−127.2	−81.8	−65.1
Binding energy	153.146	88.061	84.587	7953.5	7938.7	7924.2

*All quantities are in (k cal mol^−1^).

### Corrosion inhibition mechanism by the extract of *Terminalia bellerica* on steel

A corrosion reaction consists of two electrochemical reactions: oxidation on the anode and reduction on the cathode.

$$\mathrm{Fe }\to \mathrm{ Fe}2+ + 2\mathrm{e}-$$

$$2\mathrm{H}+ + 2\mathrm{e}- \to \mathrm{ H}2$$

When the electrons of the heteroatoms donate electrons to the empty orbital of the metal, a coordinate bond is formed^94^.

**Figure 14:**
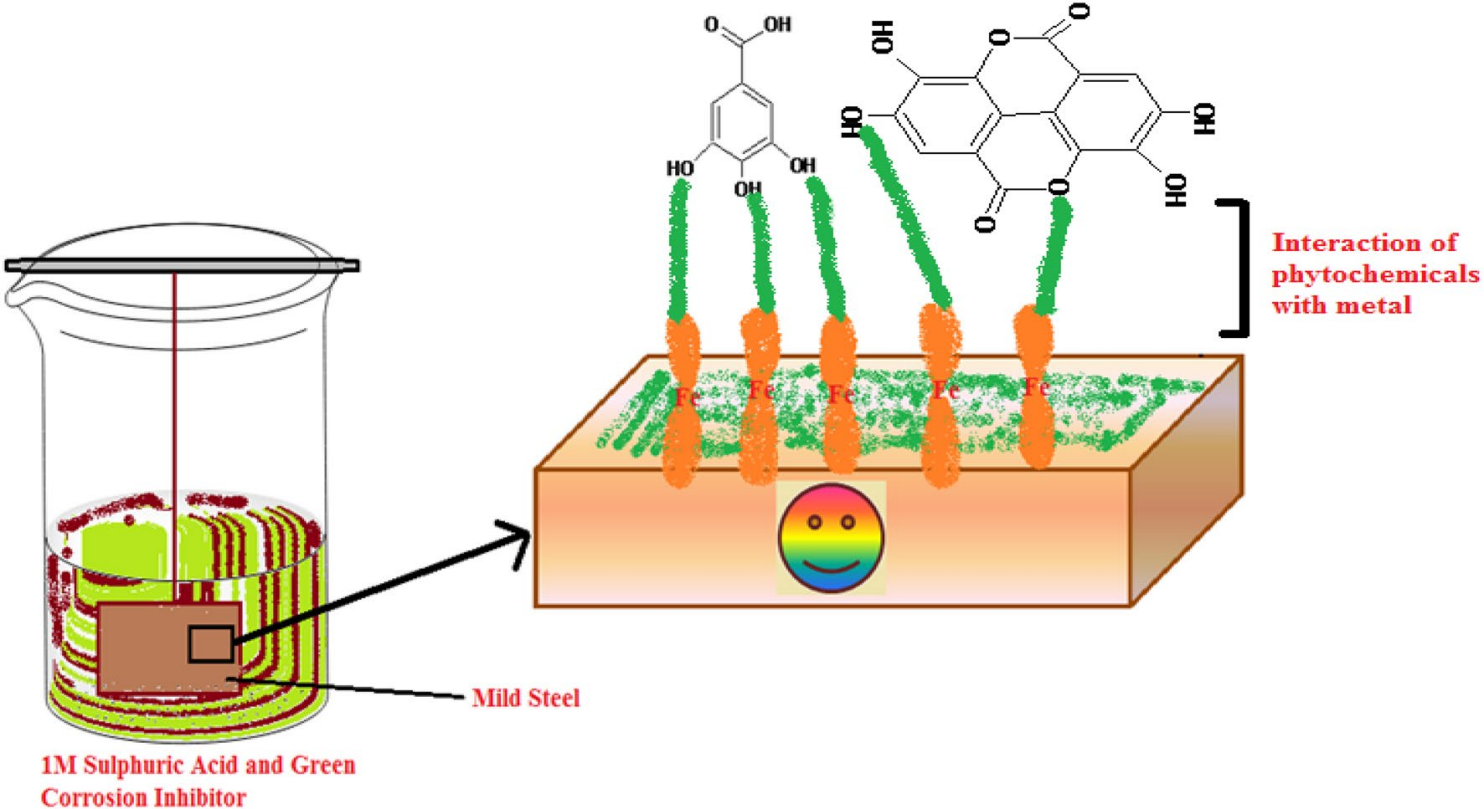
*Terminalia bellerica*’s phytochemical adsorption on steel to form protective layer.

In 1 M sulfuric acid, steel corrosion can be reduced using phytochemicals present in *Terminalia bellerica* extract. These phytochemicals have a variety of functional groups, like carbonyl, hydroxyl, and carboxylic acids, as well as double bonds. Figure 14 illustrates how the lone pair of heteroatoms and the multiple bonds found in the phytochemicals form a coordinate bond. It is the inhibitory activity of the plant extract on the metal surface that leads to blocking of the active site.

## Conclusion

1. The weight reduction method, potentiodynamic polarization study, and DFT were used to assess the corrosion resistance of steel immersed in H_2_SO_4_ in the absence of *Terminalia bellerica* extract and in presence of extract.
2. The addition of a fruit extract from *Terminalia bellerica* seems to increase the effectiveness of corrosion inhibition.
3. It has been found that at a concentration of 4000 ppm, the inhibitory efficacy was 91.79%.
4. The potentiodynamic polarization investigation shows that when the concentration of *Terminalia bellerica* extracts raises, the corrosion current density decreases.
5. The various phytochemicals found in the extract have an impact on the inhibition's potency.
6. As the inhibitor concentration is increased, the inhibition efficiency improves.
7. According to the NBO study, Ellagic acid has a stronger inhibitory efficiency due to the low energy gaps that may promote the reactivity of these molecules toward the metal surface.
8. As a result of the Langmuir adsorption isotherm, SEM, and AFM, the adsorption process has been identified as the mechanism that prevents corrosion.

### Supplementary Information

Supplementary Tables.

## Data Availability

The datasets generated during and/or analyzed during the current study are available from the corresponding author on reasonable request.
