# Supplementary material for: Electrochemical and DFT studies of Terminalia bellerica fruit extract as an eco-friendly inhibitor for the corrosion of steel
Source: Sci Rep. 2023 Nov 8;13:19367. doi: 10.1038/s41598-023-45283-0 (PMC10632492; doi:10.1038/s41598-023-45283-0)
Supplement: Supplementary file 1 — Supplementary Tables. [file 41598_2023_45283_MOESM1_ESM.docx]

**Table S1:** Calculated NBOs densities of Ellagic acid at expected adsorption sites.

| LP(2)O_27_ | LP(2)O_28_ |
| --- | --- |
| 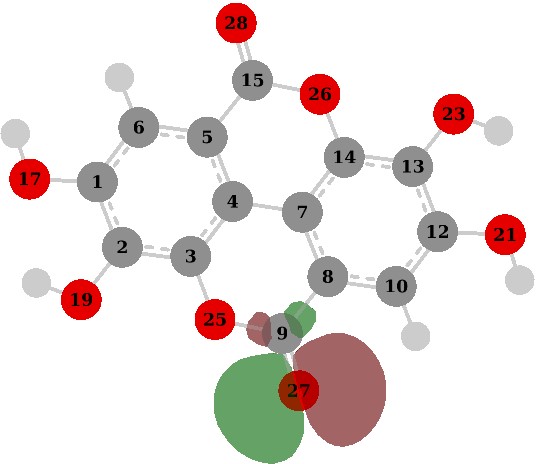 | 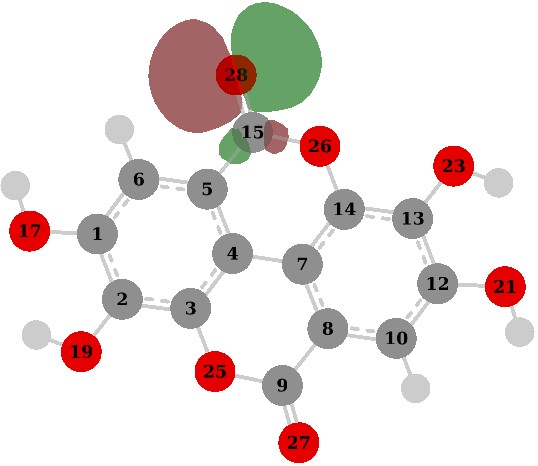 |
| BD(2)C_7_-C_8_ | BD(2)C_4_-C_5_ |
| 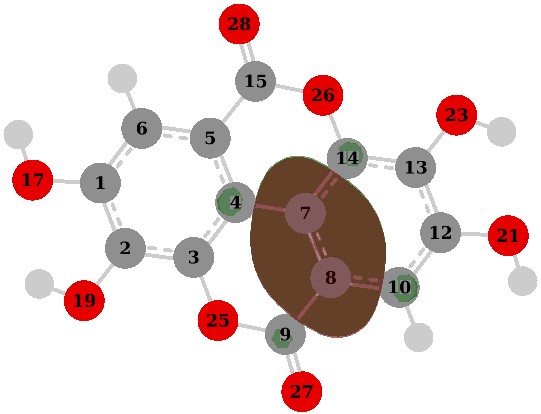 | 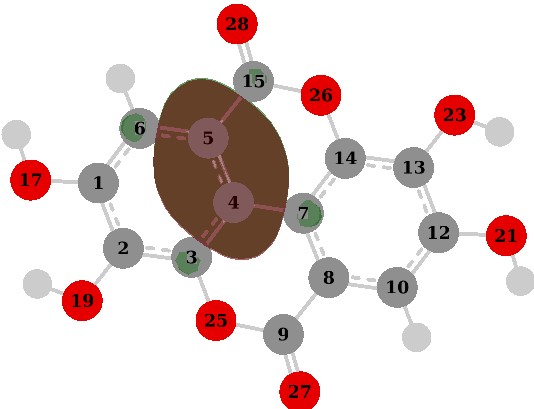 |
| BD(2)C_10_-C_12_ | BD(2)C_1_-C_6_ |
| 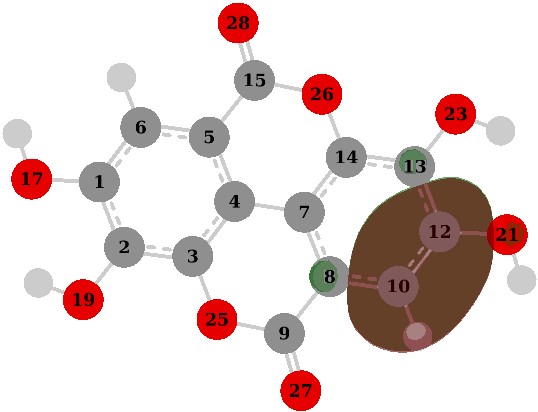 | 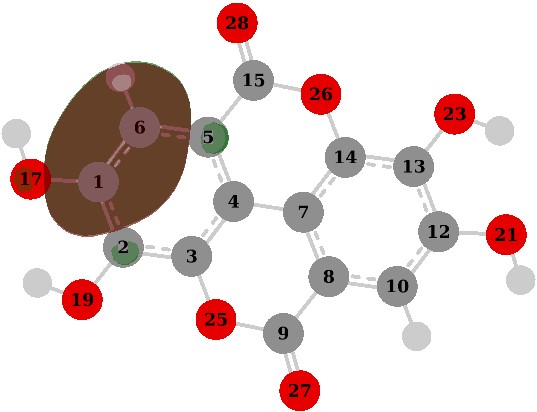 |
| BD(2)C_13_-C_14_ | BD(2)C_2_-C_3_ |
| 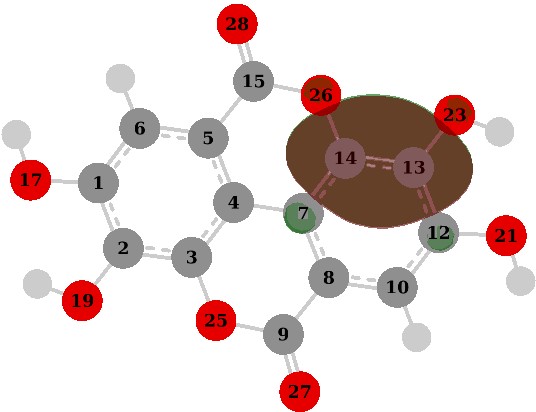 | 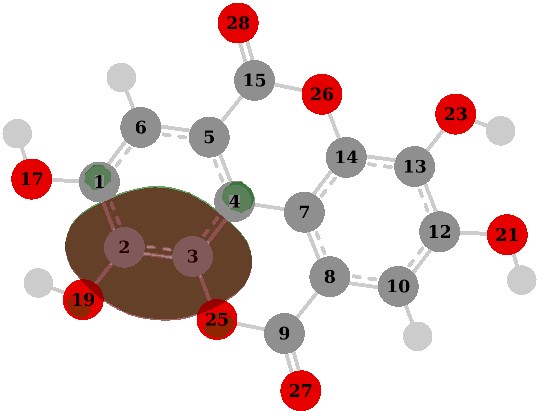 |
| LP(2)O_23_ | LP(2)O_19_ |
| 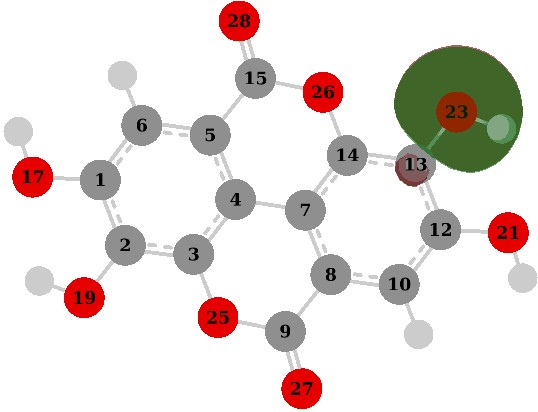 | 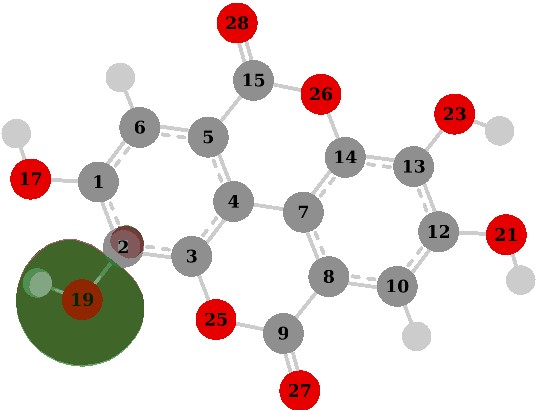 |
| LP(2)O_26_ | LP(2)O_25_ |
| 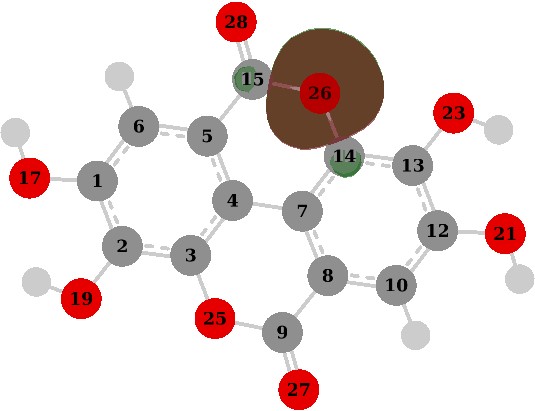 | 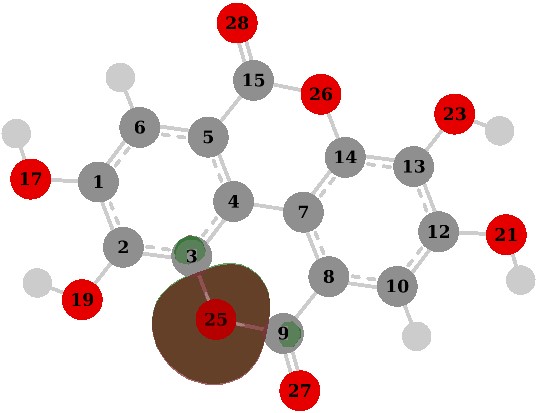 |
| LP(2)O_21_ | LP(2)O_17_ |
| 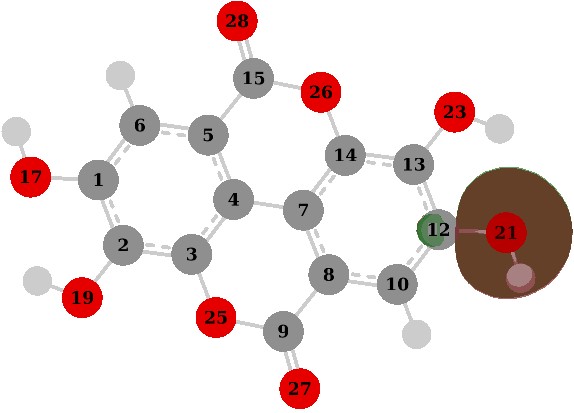 | 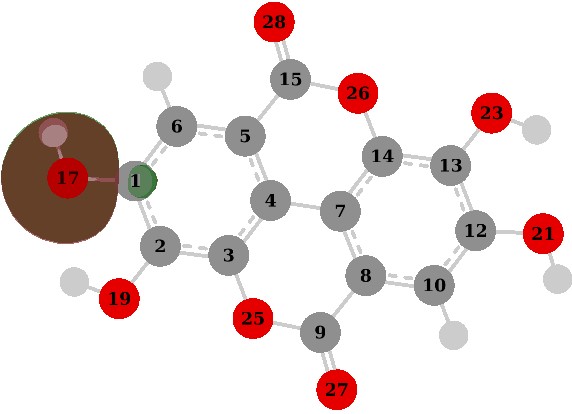 |
| BD(2)C_9_-O_27_ | BD(2)C_15_-O_28_ |
| 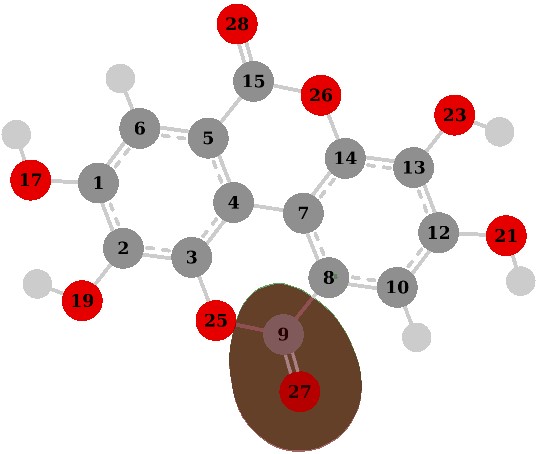 | 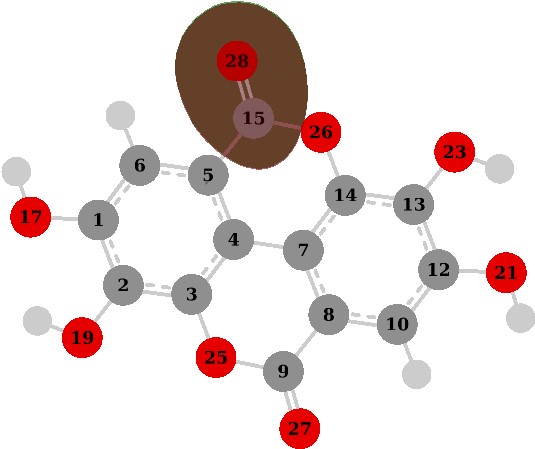 |

**Table S2:** Calculated NBOs densities of Gallic acid at expected adsorption sites.

| BD(2)C_3_-C_4_ | LP(2)O_10_ |
| --- | --- |
| 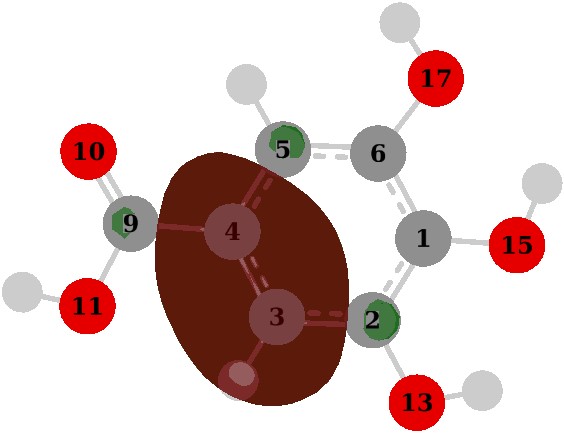 | 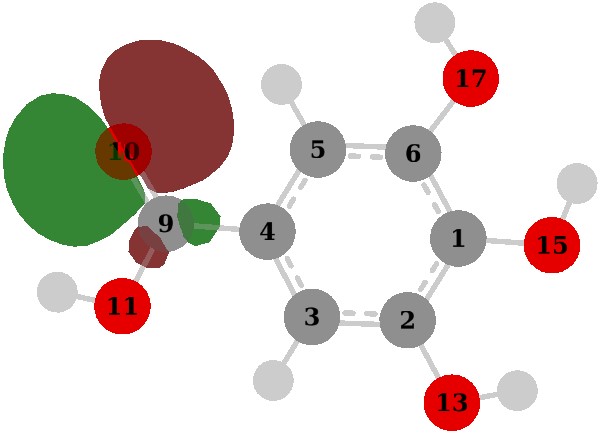 |
| BD(2)C_5_-C_6_ | BD(2)C_1_-C_2_ |
| 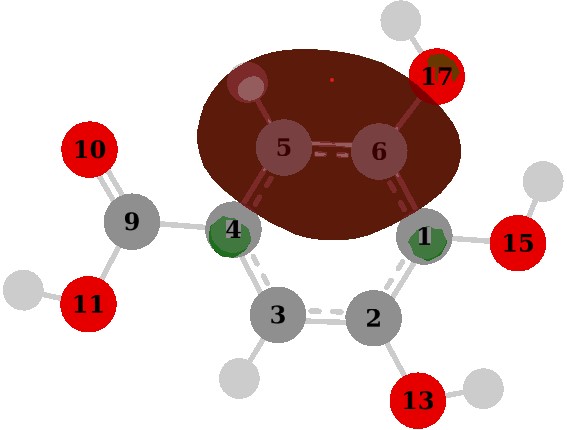 | 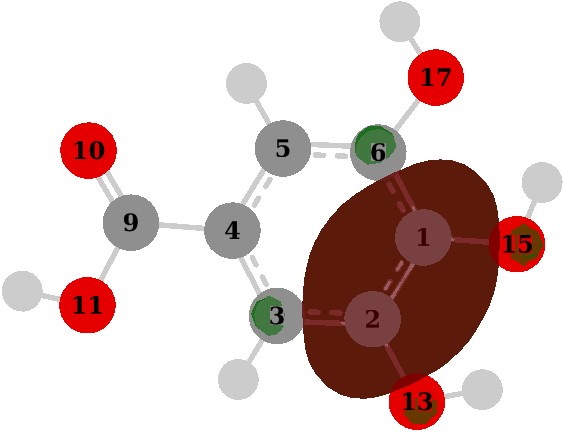 |
| LP(2)O_13_ | LP(2)O_11_ |
| 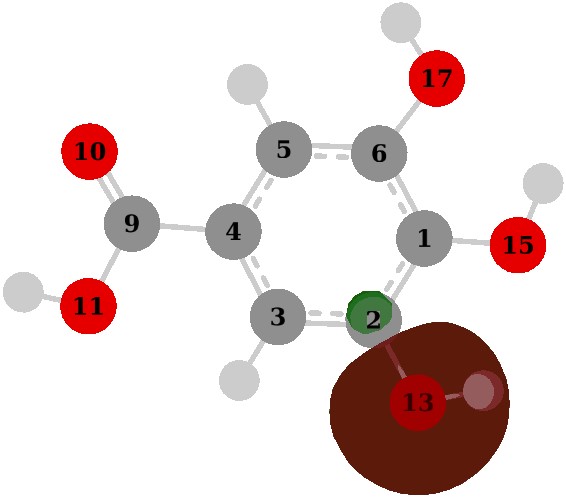 | 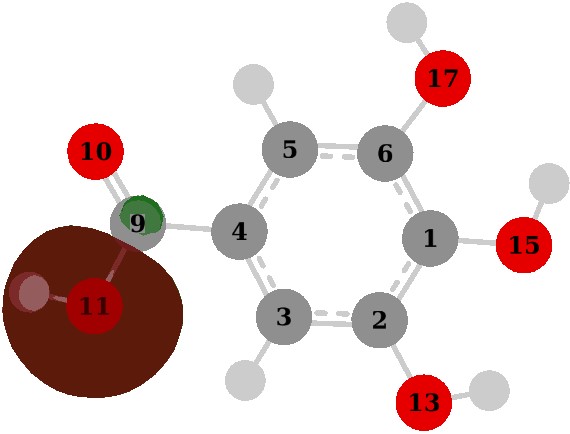 |
| LP(2)O_15_ | LP(2)O_17_ |
| 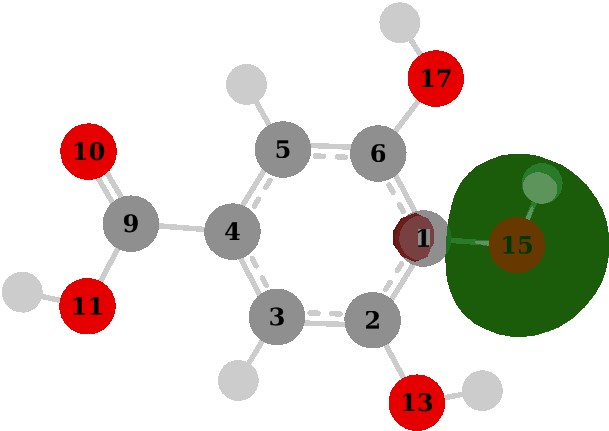 | 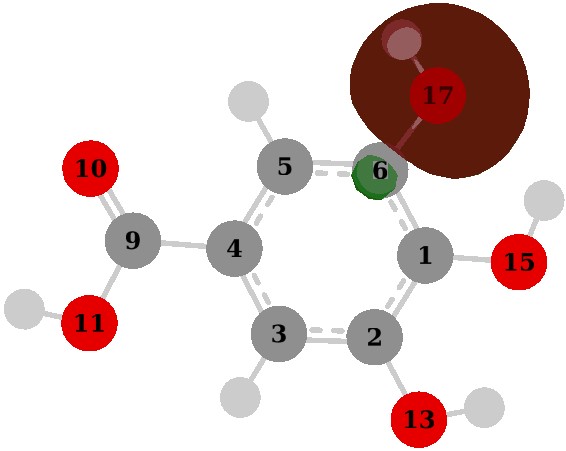 |
| BD(2)C_9_-O_10_ | BD(1)C_3_-H_7_ |
| 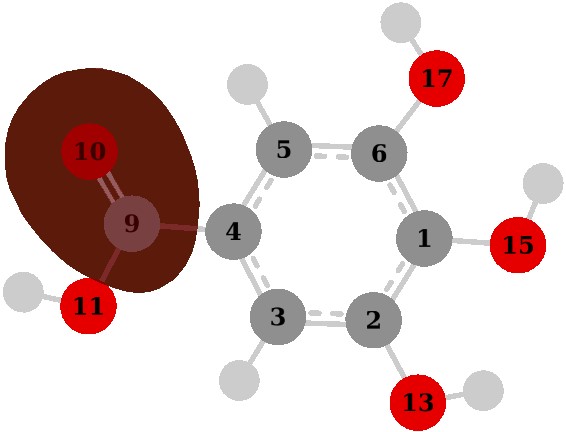 | 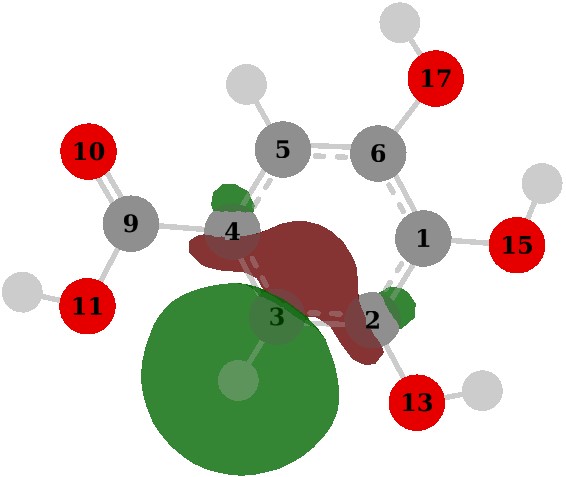 |

**Table S3:** Calculated NBOs densities of Malic acid at expected adsorption sites.

| LP(2)O_16_ | LP(2)O_10_ |
| --- | --- |
| 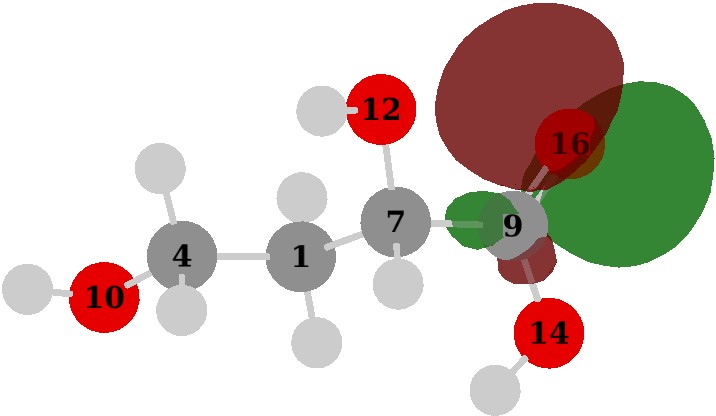 | 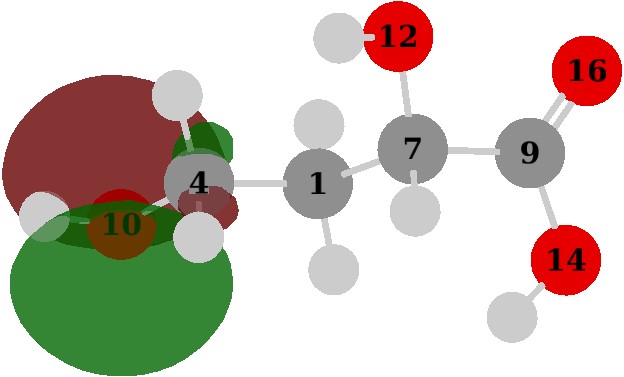 |
| LP(2)O_12_ | LP(2)O_14_ |
| 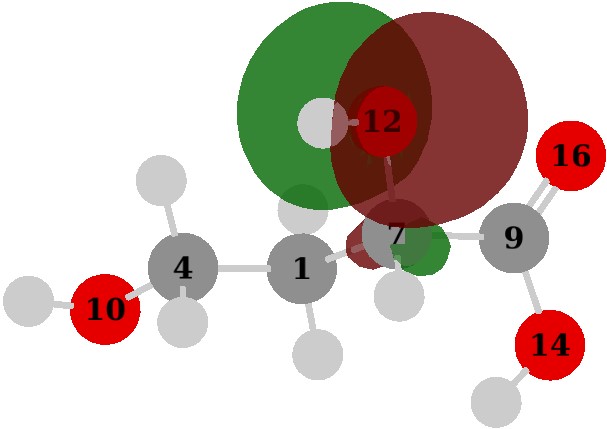 | 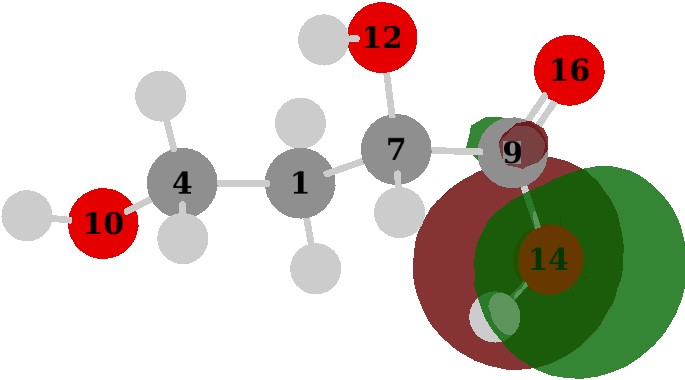 |
| BD(2)C_9_-O_16_ | BD(1)C_1_-H_2_ |
| 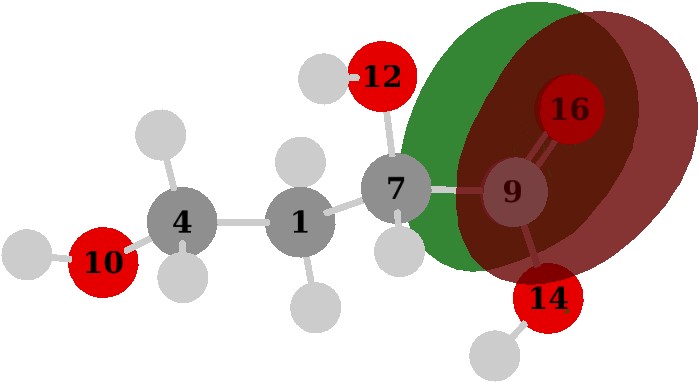 | 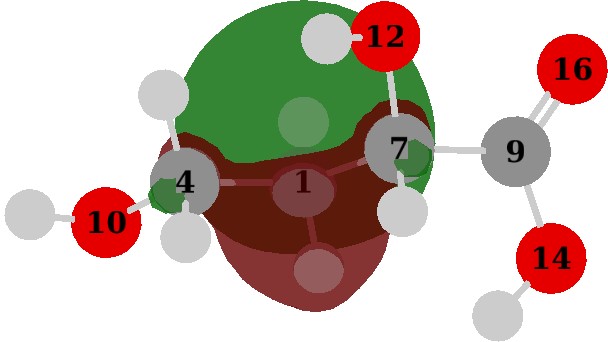 |
